# Initial Blood pH, Lactate and Base Deficit Add No Value to Peri-Arrest Factors in Prognostication of Neurological Outcome After Out-of-Hospital Cardiac Arrest

**DOI:** 10.3389/fmed.2021.697906

**Published:** 2021-09-16

**Authors:** Matthias Mueller, Juergen Grafeneder, Christian Schoergenhofer, Michael Schwameis, Christoph Schriefl, Michael Poppe, Christian Clodi, Moritz Koch, Fritz Sterz, Michael Holzer, Florian Ettl

**Affiliations:** ^1^Department of Emergency Medicine, Medical University of Vienna, Vienna, Austria; ^2^Department of Clinical Pharmacology, Medical University of Vienna, Vienna, Austria

**Keywords:** cardiac arrest, resuscitation, blood gas analysis, blood pH, lactate, base deficit, outcome prediction, peri-arrest factors

## Abstract

**Background:** In cardiac arrest survivors, metabolic parameters [pH value, lactate concentration, and base deficit (BD)] are routinely added to peri-arrest factors (including age, sex, bystander cardiopulmonary resuscitation, shockable first rhythm, resuscitation duration, adrenaline dose) to enhance early outcome prediction. However, the additional value of this strategy remains unclear.

**Methods:** We used our resuscitation database to screen all patients ≥18 years who had suffered in- or out-of-hospital cardiac arrest (IHCA, OHCA) between January 1st, 2005 and May 1st, 2019. Patients with incomplete data, without return of spontaneous circulation or treatment with sodium bicarbonate were excluded. To analyse the added value of metabolic parameters to prognosticate neurological function, we built three models using logistic regression. These models included: (1) Peri-arrest factors only, (2) peri-arrest factors plus metabolic parameters and (3) metabolic parameters only. Receiver operating characteristics curves regarding 30-day good neurological function (Cerebral Performance Category 1-2) were analysed.

**Results:** A total of 2,317 patients (OHCA: *n* = 1842) were included. In patients with OHCA, model 1 and 2 had comparable predictive value. Model 3 was inferior compared to model 1. In IHCA patients, model 2 performed best, whereas both metabolic (model 3) and peri-arrest factors (model 1) demonstrated similar power. PH, lactate and BD had interchangeable areas under the curve in both IHCA and OHCA.

**Conclusion:** Although metabolic parameters may play a role in IHCA, no additional value in the prediction of good neurological outcome could be found in patients with OHCA. This highlights the importance of accurate anamnesis especially in patients with OHCA.

## Introduction

Sudden cardiac arrest is a global health burden with an incidence of 86.4/100.000 person-years in Europe (1). With a survival rate of only 10%, and an enormous risk of cerebral damage, tools for early prognostication are desperately needed (2).

Peri-arrest factors associated with poor neurological outcome after cardiopulmonary resuscitation (CPR) include unwitnessed arrest, no bystander resuscitation, longer duration to return of spontaneous circulation (ROSC), non-shockable rhythms, higher cumulative doses of adrenaline (epinephrine) and increasing age (3–5). However, the early determination of these data is challenging and prone to errors, because the information commonly comes from distressed witnesses. Thus, clinicians tend to rely more on objective metabolic parameters including pH value, lactate levels and base deficit (BD) measured in the blood sample drawn on admission.

Blood gas analysis is recommended during and after CPR to identify electrolyte imbalances and acidaemia (6). High lactate levels on hospital admission are associated with poor survival and poor neurological outcome after sudden cardiac arrest (7, 8). A meta-analysis from Zhou et al. revealed that initial lactate has a better predictive value for neurological outcome than lactate clearance during the post-CPR course (9).

Due to their nature, pH, lactate and BD interact to some extent, although after resuscitation different pathomechanisms may contribute to each parameter. Blood pH is affected by both respiratory and metabolic disorders. Although Weil et al. found that arterial blood pH values do not reflect the pH value in the tissue properly during CPR (10), a recently published study by Carr et al. found that pH values predicted survival to hospital discharge (11). BD is influenced by all unmeasured anions (lactate, uraemia, ketone bodies etc.) and deviation is associated with poor neurological outcomes (12, 13). In trauma patients, admission BD is superior to lactate in the prediction of resuscitation needs and mortality (14). Funk et al. (15) showed that higher amounts of unmeasured anions are associated with poor neurological survival after cardiac arrest.

Several scores, including both metabolic parameters and peri-arrest factors, were tested in cardiac arrest patients (16–18). Interestingly, no data is available on the added value of metabolic parameters to peri-arrest factors. We hypothesised that metabolic parameters may serve to confirm the clinical impression of the medical team in charge, but fail to add value to prognostication. Therefore, we investigated the added value of metabolic parameters to peri-arrest factors in the prediction of neurological outcome after sudden cardiac arrest.

## Materials and Methods

### Study Design and Population

This study analysed prospectively collected data from the Emergency Department of the Medical University of Vienna's resuscitation database. Detailed information about this database is given elsewhere (19). Briefly, all patients with in- and out of hospital cardiac arrest (IHCA, OHCA) treated at our facility were prospectively included, and were followed up for 12 months. Data acquisition was performed by specially trained members of the resuscitation research group in accordance with the Utstein style (20).

All patients ≥18 years of age who suffered an IHCA or OHCA between January 1st, 2005 and May 1st, 2019 were included in our analysis. Patients who never achieved ROSC or were treated with sodium bicarbonate were excluded. We also excluded patients with incomplete data set. “Bystander CPR” was defined as “CPR by a person who is not responding as part of an organised emergency response system to a cardiac arrest” according to the Utstein guidelines (20). “Advanced live support (ALS) initially” was defined as ALS from 0 to 2 min after arrest. For model analyses, bystander CPR and ALS initially were grouped together and called “initial CPR” and then compared with “no initial CPR”.

Blood samples were taken via femoral or radial arterial access immediately after admission (OHCA) or after ROSC (IHCA) respectively. In case of admission with ongoing CPR, blood gas analyses were drawn immediately after ROSC. Analyses were performed at the bedside using the ABL800 Flex blood gas analyser (Radiometer Medical ApS, Brønshøj, Denmark). Together with blood gas analysis, routine laboratory samples were collected and sent to the hospital's certified laboratory.

### Setting

The Medical University of Vienna's Emergency Department provides the full spectrum of intensive care medicine and is located at the General Hospital of Vienna, a tertiary care centre that treats approximately half of all OHCA patients in the metropolitan area. Furthermore, the department dispatches a medical emergency team within the hospital. Coronary angiography ± PCI, extracorporeal membrane oxygenation and cardiothoracic surgery are available at all times. Out of hospital care is performed by the Vienna municipal emergency medical service (EMS), a physician based system operating with a mean time to first medical contact of 7 ± 3 min (21).

After pre-alert from the emergency medical service (EMS) dispatcher, a member of the resuscitation research group immediately contacts the EMS coordinating centre to get additional data (e.g., time of alert, time of arrival of the first EMS unit, telephone assisted CPR, units at scene, and callback number). After this, staff on scene and any witnesses are called for additional information (prodromal symptoms, preexisting conditions, performance of bystander CPR, first rhythm, estimated no- and low flow time, patient's insurance data). Thereafter, the electronic patient records are screened for additional information from previous hospital visits. The findings are documented in a standardised manner (20, 22). By applying these steps, we aim to have the best possible anamnesis and patient history available, even before the patient arrives at our department. The recorded data may further influence the clinical treatment (extracorporeal cardiopulmonary resuscitation, pre-alert of the coronary angiography laboratory).

### Models

We used a logistic regression with the neurological outcome dichotomized. We selected covariables based on previous studies and clinical reasoning for the development of our models (23, 24). Model 1 included the following parameters: age (in years), sex (male/female), initial CPR (yes/no), initial rhythm shockable (yes/no), witness status (yes/no), adrenaline dosage (cumulative dosage in mg). Model 2 included all parameters in model 1 with the addition of the following parameters: blood pH level, lactate level (mmol/L), base deficit (mmol/L). Model 3 includes only the metabolic parameters (blood pH, lactate levels, and base deficit). All patients were included in the models. Collinearity of independent variables was tested through calculation of variance inflation factors before running the multivariable regression analysis. No significant collinearity of independent variables was detected (calculations can be found in the Supplementary Material).

### Statistical Analysis

Categorical variables are summarised as counts (n) and frequencies (%), continuous variables are expressed as mean and standard deviation (SD) or median and interquartile range (IQR), as applicable.

Missing data were reported with missing plots and a missing pattern figure in the Supplementary Material.

The primary endpoint was defined as the difference in performance between model 1 (peri-arrest factors) and model 2 (peri-arrest factors plus metabolic parameters) in the prediction of good neurological outcome at 30 days. Good neurological outcome was defined as Cerebral Performance Category (CPC) 1-2. For the analyses, we dichotomized the CPC (good outcome: CPC 1-2, bad outcome: CPC 3-5).

The performance was assessed through a receiver operating characteristic analysis. Area under the curve (AUC) with a 99% CI and *p*-value are reported. We used the method by DeLong et al. to compare AUCs (25).

As secondary endpoints, we analysed the crude performance of blood pH, lactate levels and base deficit to predict good neurological outcome (CPC 1-2) at 30 days. We further compared the metabolic parameters alone (model 3) vs. our peri-arrest factors outcome model (model 1). The model calculations can be found in the Supplementary Material.

Given the large sample size, a two-sided *p* < 0.01 was considered statistically significant in accordance to the formula of Good and Lakens to avoid a type I error (26–30).

The models are reported according to the TRIPOD statement (31). For sensitivity analyses, we calculated multiple imputations for missing data using chained equations (R-package “mice”; see Supplementary Material).

### Ethical Approval

This study was approved by the local ethics committee (EK No. 1219/2018) and complies with the declaration of Helsinki.

## Results

**Figure 1:**
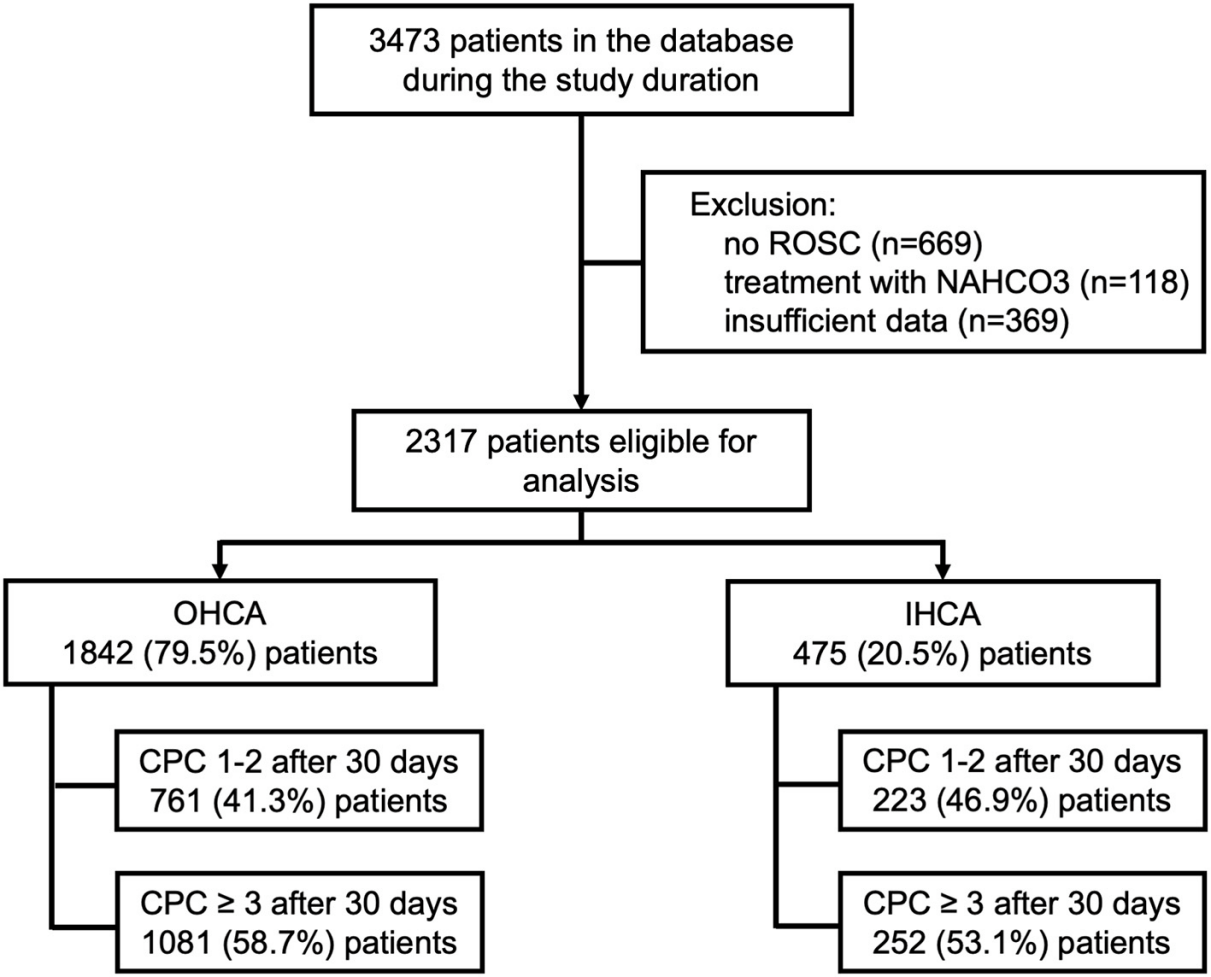
Study flowchart.

During the study period (January 1st, 2005 and May 1st, 2019), 3473 patients were treated at our facility following cardiac arrest. After exclusion of 1156 patients, 2317 patients remained eligible for analysis (Figure 1). A total of 475 (20.5%) patients were resuscitated from IHCA, whereas 1842 (79.5%) patients were treated after OHCA.

Patients with IHCA were older (67 vs. 60 years, *p* < 0.001), more often female (33.7 vs. 27.4%, *p* = 0.007), had a higher rate of diabetes (25.9 vs. 17.9%, *p* < 0.001) and a lower CPC 1 rate prior to the event (82.1 vs. 89.3%, *p* < 0.001). Their arrests were more often witnessed (94.9 vs. 83.0%, *p* < 0.001) with higher numbers of bystander CPR or initial ALS (97.3 vs. 72.3%, *p* < 0.001) resulting in a shorter duration of resuscitation (6 vs. 22 min, *p* < 0.001) and a smaller cumulative adrenaline dose (1 vs. 2 mg, *p* < 0.001). However, shockable initial rhythms (34.5 vs. 57.7%, *p* < 0.001) and cardiac aetiology (58.9 vs. 64.7%, *p* < 0.001) occurred less frequently. IHCA was more frequently caused by respiratory diseases (18.1 vs. 13.0%, *p* = 0.004) and other noncardiac reasons (7.8 vs. 2.2%, *p* < 0.001).

In both IHCA and OHCA, patients with CPC 3-5 had worse peri-arrest factors. Details are shown in Table 1.

**Table 1:** Baseline characteristics according to outcome at 30 days after admission.

	**Out-of-hospital cardiac arrest (** ***n*** **= 1,842)**		**In-hospital cardiac arrest (** ***n*** **= 475)**	
	**CPC 1-2** **(*n* = 761)**	**CPC 3-5** **(*n* = 1,081)**	**CPC 1-2** **(*n* = 223)**	**CPC 3-5** **(*n* = 252)**
Age	56 [47–66]^*^	63 [51–73]	65 [51–74]^*^	69 [58–78]
Female sex	179 (23.5)^*^	326 (30.2)	74 (33.2)	86 (34.1)
BMI	26.2 [24.2–29.3]	26.2 [24.2–30.1]	26.5 [23.4–30.4]	26.1 [23.4–29.4]
Diabetes	93 (12.2)^*^	236 (21.8)	51 (22.9)	72 (28.6)
COPD	71 (9.3)^*^	163 (15.1)	27 (12.1)	34 (13.5)
CPC 1 prior to event	715 (94.0)^*^	929 (85.9)	198 (88.8)^*^	192 (76.2)
Witnessed arrest	688 (90.4)^*^	841 (77.8)	215 (96.4)	236 (93.7)
Bystander CPR	440 (57.8)^*^	542 (50.1)	34 (15.2)	47 (18.7)
ALS initially	187 (24.6)^*^	163 (15.1)	187 (83.9)	194 (77.0)
Resuscitation length in min.	16 [10–24]^*^	29 [19–44]	4 [1–11]^*^	8 [3–18]
Adrenaline in mg	0.3 [0–2]^*^	3 [1–4.5]	0 [0–1]^*^	1.2 [1–3]
Initially shockable	585 (76.9)^*^	477 (44.1)	110 (49.3)^*^	54 (21.4)
**ETIOLOGY**				
Presumed cardiac	601 (79.0)^*^	591 (54.7)	150 (67.3)^*^	130 (51.6)
Trauma	2 (0.3)	3 (0.3)	1 (0.4)	2 (0.8)
Drowning	7 (0.9)	20 (1.9)	0	0
Respiratory	52 (6.8)^*^	187 (17.3)	30 (13.5)	56 (22.2)
Other noncardiac	40 (5.3)^*^	165 (15.3)	28 (12.6)	45 (17.9)
Unknown	59 (7.8)	115 (10.6)	14 (6.3)	19 (7.5)

* *statistically significant differences (p < 0.01) between good (cerebral performance category–CPC 1-2) and bad (CPC ≥3) neurological outcome at 30 days after cardiac arrest. ALS, advanced life support; BMI, Body mass index; COPD, Chronic obstructive pulmonary disease; CPR, cardiopulmonary resuscitation; mg, milligrams, min: minutes*.

The results from the first blood gas analysis after admission to the emergency department are shown in Table 2. Creatinine and albumin levels were measured in the central laboratory (blood sampling was done at the same time).

**Table 2:** Blood gas analysis values at emergency department admission after in- and out of hospital cardiac arrest regarding good (cerebral performance category–CPC 1-2) and bad (CPC ≥3) neurological outcome at 30 days after cardiac arrest.

	**Out-of-hospital cardiac arrest**		**In-hospital cardiac** **arrest**	
	**CPC 1-2**	**CPC 3-5**	**CPC 1-2**	**CPC 3-5**
**BLOOD GAS ANALYSIS AND INITIAL LAB VALUES AT ADMISSION**				
pH	7.240 [7.142–7.315]	7.115 [6.962–7.222]	7.322 [7.180–7.393]	7.154 [6.994–7.269]
pCO2 in mmHg	46.3 [40.8–55.5]	51.1 [41.3–66.0]	41.5 [33.9–52.8]	47.5 [37.9–60.3]
pO2 in mmHg	154.5 [89.7–342.0]	172.0 [97.2–369.0]	111 [75.6–177.0]	112 [77.9–222.0]
Potassium in mmol/L	3.7 [3.3–4.1]	4 [3.5–4.6]	3.9 [3.5–4.3]	4.2 [3.7–4.9]
Lactate in mmol/L	5.5 [3.2–8.2]	9.2 [6.3–12.5]	3.8 [2.1–7.3]	7.6 [4.8–11.3]
Base deficit in mmol/L	−6.7 [−10.4–3.7]	−12.1 [−16.7–7.7]	−4.5 [−9.5–1.0]	−10.5 [−16.0–5.7]
Hydrogen carbonate in mmol/L	18.2 [15.1–20.9]	14.1 [10.5–17.3]	20.4 [16.2–23.2]	15.2 [11.7–19.0]
Creatinine in mg/dL	1.18 [0.99–1.39]	1.38 [1.15–1.67]	1.1 [0.91–1.40]	1.48 [1.08–2.23]
Albumin in g/L	38.3 [35.3–40.8]	35.3 [31.5–38.6]	36.7 [31.0–40.4]	31.2 [26.4–36.8]

*All comparisons between CPC 1-2 and CPC 3-5 statistically significant (p < 0.01) with the exception of pO2*.

**Figure 2:**
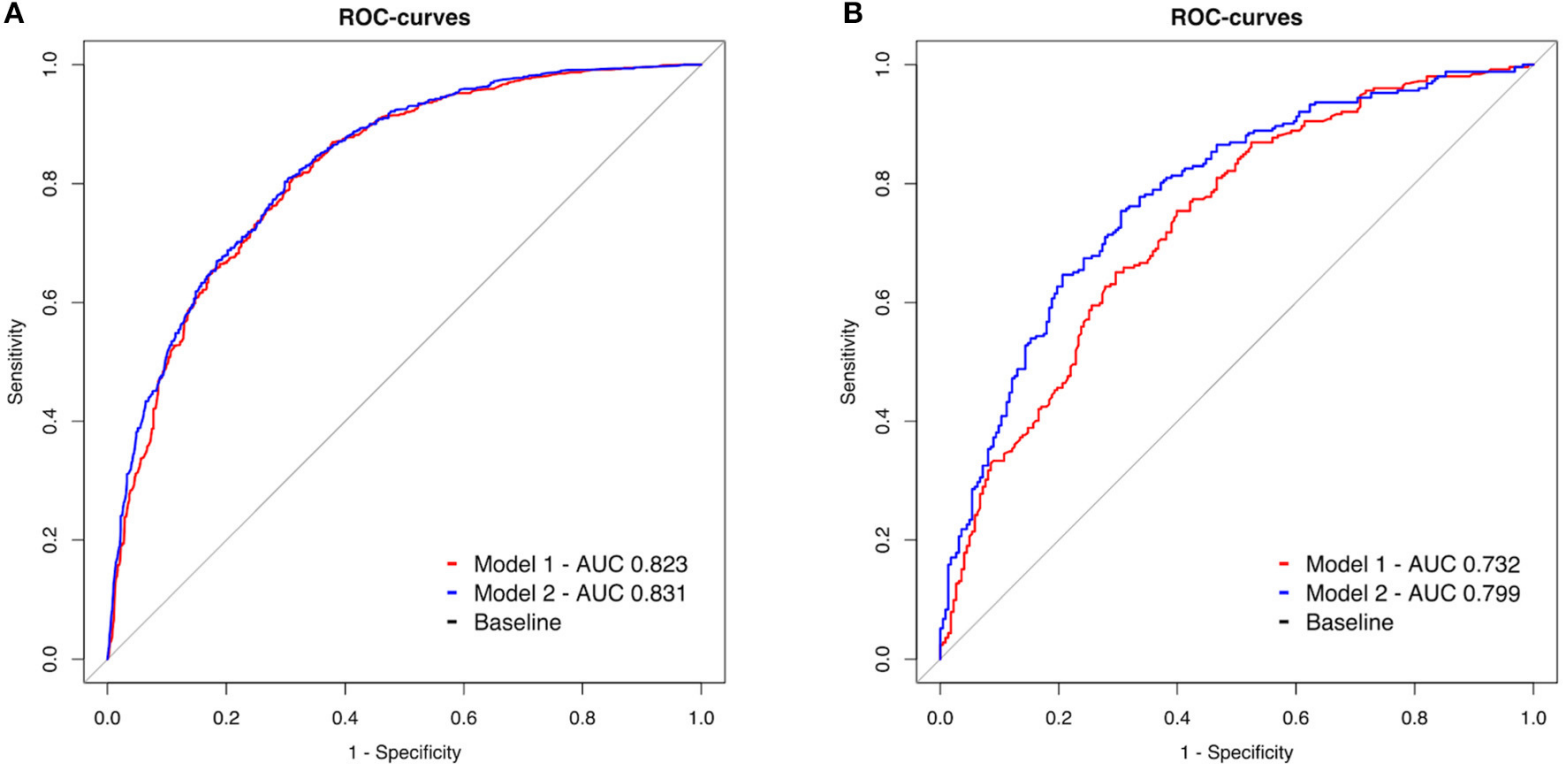
**(A,B)** Receiver operating characteristic (ROC) curves of two models to predict 30-day neurological outcome. Model 1 (red line): Inclusion of peri-arrest factors (age, sex, witness status, and BLS, shockable initial rhythm, cumulative adrenaline). Model 2 (blue line): model 1 combined with the parameters from the initial blood gas analysis (pH, lactate level, and base deficit). OHCA: out of hospital cardiac arrest, IHCA: in hospital cardiac arrest.

Figure 2 shows the results of the ROC analysis for model 1 and 2 for OHCA and IHCA patients. In OHCA patients, the AUC of model 1 (0.823, 99% CI 0.797–0.848) and model 2 (0.831, 99% CI 0.806–0.855) did not differ significantly (*p* = 0.013, Figure 2A). In IHCA patients, there was a significant difference between model 1 (AUC 0.732, 99% CI: 0.673–0.791) and model 2 (AUC 0.779 99% CI 0.725–0.834, *p* < 0.001, Figure 2B).

**Figure 3:**
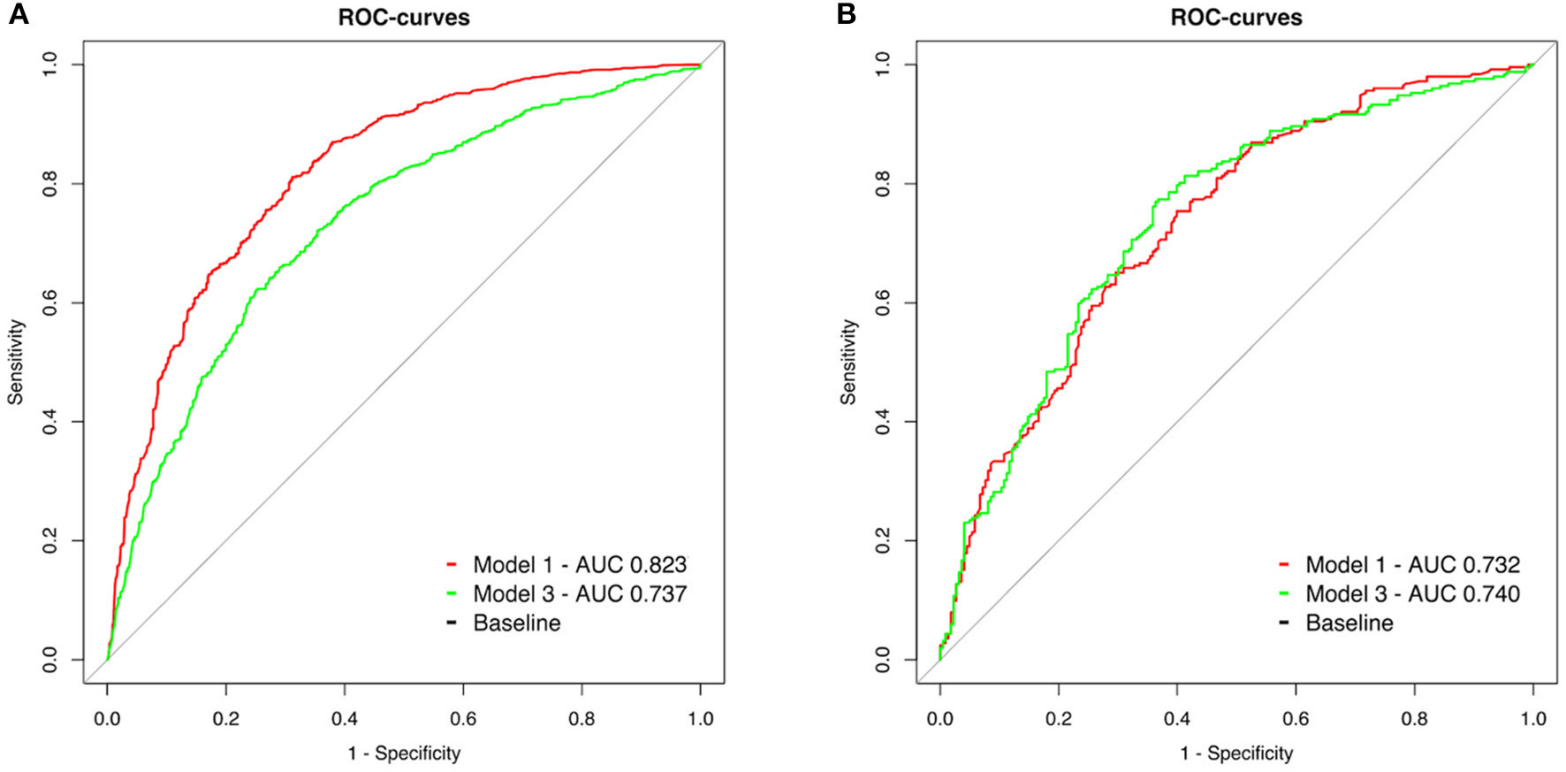
**(A,B)** Receiver operating characteristic (ROC) curves of two models to predict 30-day neurological outcome. Model 1 (red line): Inclusion of peri-arrest factors (age, sex, witness status, BLS, shockable initial rhythm, and cumulative adrenaline). Model 3 (green line): Metabolic parameters from the initial blood gas analysis (pH, lactate level, and base deficit).

The peri-arrest factors model (model 1) demonstrated better sensitivity and specificity than pH, lactate and BD (model 3) without overlap in 99% confidence intervals in OHCA (model 1: AUC 0.823, 99% CI 0.797–0.848, model 3: AUC 0.737, 99% CI 0.706–0.767, *p* < 0.001, Figure 3A).

In IHCA, the predictive value of blood gas parameters was similar to peri-arrest factors (model 1: AUC 0.732, 99% CI 0.673–0.791, model 3: AUC 0.740 99% CI 0.681–0.799, *p* = 0.77, Figure 3B).

**Figure 4:**
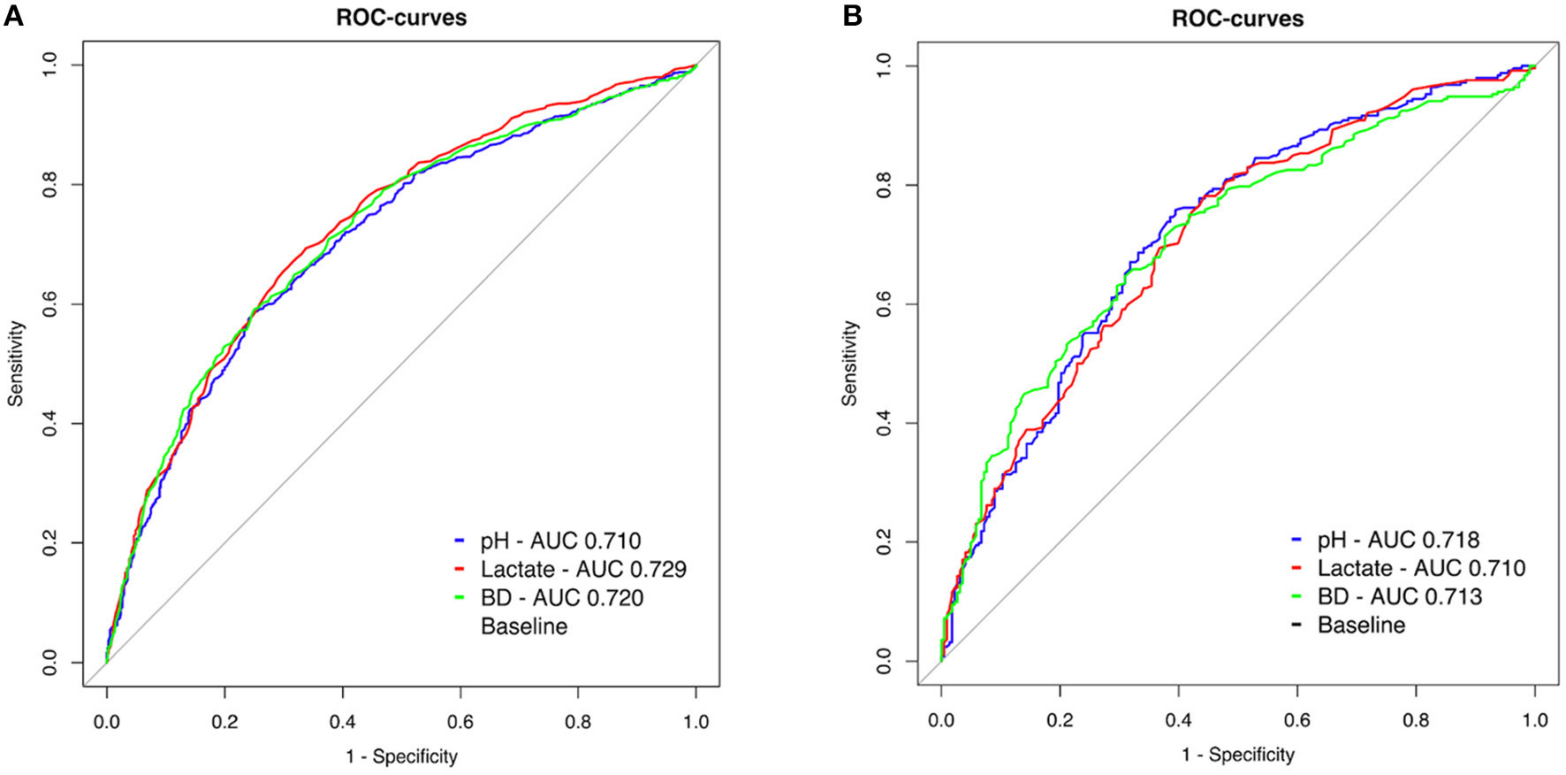
**(A,B)** Receiver operating characteristic (ROC) curves of pH (blue), lactate (red) and base deficit (green) at admission to predict 30-day neurological outcome.

We further compared the predictive value of pH, lactate and BD separately. In OHCA, the AUC of receiver operating curves were comparable for pH (AUC 0.710, 99% CI 0.679–0.741), lactate (AUC 0.729, 99% CI 0.699–0.760) and BD (AUC 0.720, 99% CI 0.689–0.751) (pH vs. BD *p* = 0.19, pH vs. lactate *p* = 0.03, lactate vs. BD *p* = 0.22, Figure 4A).

Also in IHCA, pH (AUC 0.718 99% CI 0.657–0.778), lactate (AUC 0.710 99% CI 0.649–0.771) and BD (AUC 0.713 99% CI 0.652–0.774) were of similar predictive value regarding CPC 1-2 after 30 days (pH vs. BD *p* = 0.79, pH vs. lactate *p* = 0.70, lactate vs. BD *p* = 0.88, Figure 4B).

**Figure 5:**
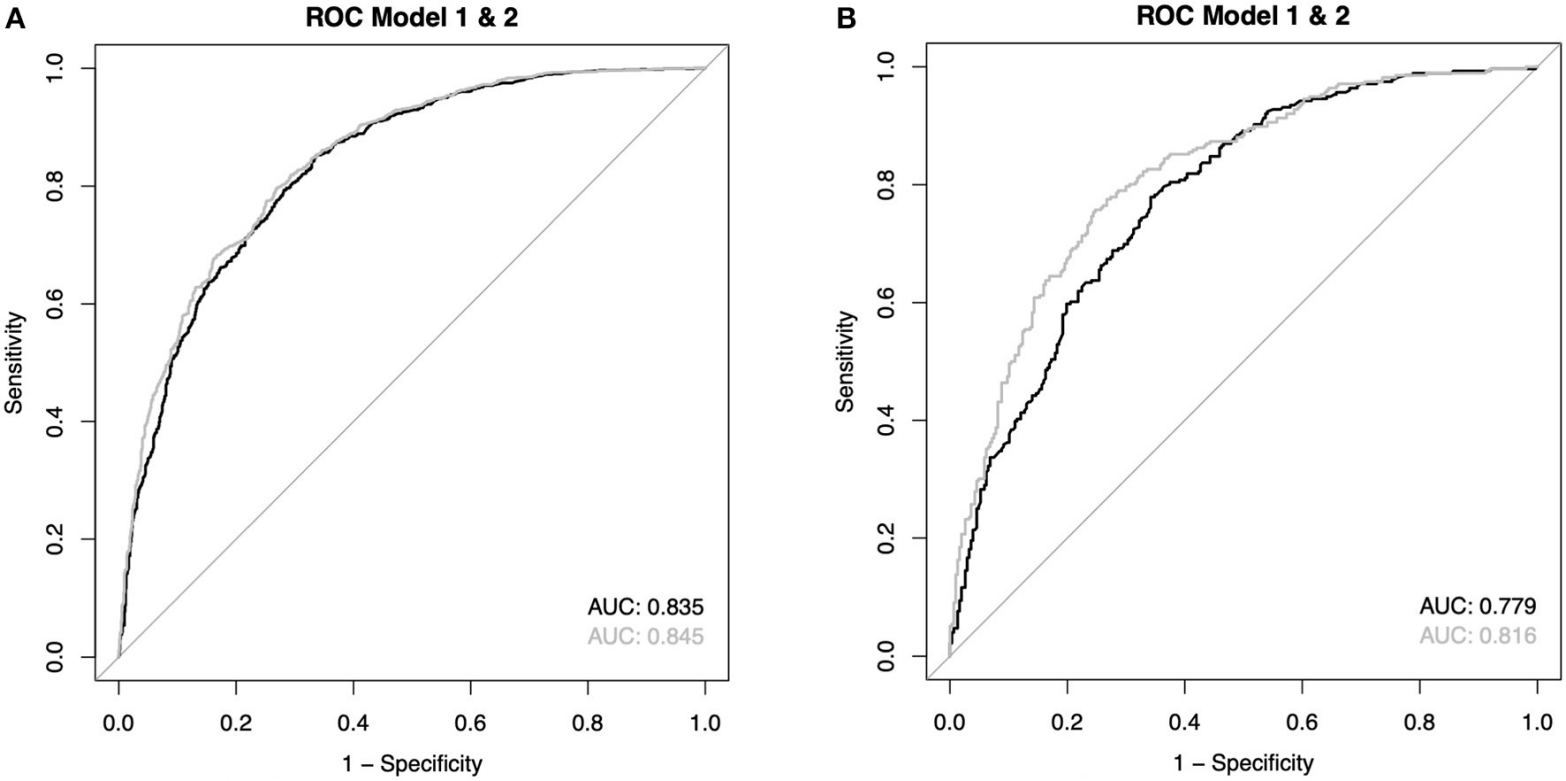
**(A,B)** Reveicer operating characteristic (ROC) curves of the average values from the imputations (see also Supplementary Material).

Multiple imputations for the missing variables were performed. The results can be found in the supplement and are comparable to the complete case analysis. The average AUC for OHCA patients model 1 was 0.835 and for model 2 0.845, respectively. After Bonferroni correction for multiple testing, two of the five logistic regressions reached a statistically significant result. However, given the average absolute difference of 0.010 for the AUCs, a clinically relevant effect seems highly unlikely. For IHCA, the AUC differed more (model 1: 0.779 vs. model 2: 0.816). The average absolute difference between the AUCs was 0.037, all *p*-values were below 0.001. The ROC curves depicting the average AUC for IHCA and OHCA can be found in Figure 5.

## Discussion

The main finding of our analysis is that the addition of metabolic parameters (i.e., blood pH, lactate and BD) to peri-arrest factors adds no value to early outcome prediction after successful resuscitation from OHCA. Furthermore, the predictive power between pH, lactate and BD is similar but nonetheless weaker than prediction based on peri-arrest factors alone. Therefore, it does not appear reasonable to use metabolic parameters alone as a predictive tool after OHCA. In contrast, after IHCA, the addition of metabolic parameters improved the prediction model and therefore may help to identify patients with an unfavourable outcome.

### Limitations of Metabolic Parameters

The measurement of metabolic parameters and a frequently unchallenged trust in objective test results presented as precise numbers may misguide clinicians in their decision making. In fact, these parameters are a highly variable composite measure reflecting several aspects of a resuscitated patient: first, the health status prior to arrest (sepsis, shock, kidney failure, hypoalbuminemia etc.); (32) second, the state and duration of no blood flow after cardiac arrest; third, the quality and duration of resuscitation delivered through bystander CPR and ALS; (33) fourth, the “resuscibility” of a patient (cardiac output generated by chest compressions) and fifth, the duration and metabolic recovery during transportation. However, it is not possible to determine to which extent the single aspects influence the test results in the initial blood gas sample.

In patients with IHCA, respiratory diseases and other noncardiac aetiologies (sepsis, metabolic derangements, and hypovolemia) occurred more often. Almost all patients immediately received bystander CPR or ALS and had a shorter length of resuscitation. Moreover, transportation times are short in hospital, which may reduce the impact of lactate clearance on the metabolic results obtained at Emergency Department admission. Thus, metabolic derangement may predominantly reflect the prior health status and resuscibility of an individual and is therefore predictive for neurological outcome.

In line with former studies, cardiac causes were the main reason for OHCA in our cohort (34). The majority of these patients collapsed from a state of subjective wellbeing. Thus, no relevant metabolic derangement is expected prior to cardiac arrest. Furthermore, diverse cardiac output and variable hospitalisation times may distort the metabolic levels after ROSC. This high variability limits the explanatory power of metabolic parameters.

### Proper History Taking

All resuscitation aspects mentioned above can be evaluated via telephone interviews with witnesses and EMS-teams on scene. Witnesses can aid in clarifying prior health status, witness state, bystander CPR and duration of no-flow time. Treating teams on scene can inform of CPR progress, initial and current heart rhythm as well as endtidal CO_2_, which are surrogate parameters for cardiac output (35, 36). Moreover, proper anamnesis allows more differentiated insights into that particular resuscitation.

Current resuscitation guidelines give no recommendations for the preparation of cardiac arrest centres between alert and patient admission (37). At our department, comprehensive history taking starts before the patient arrives (see Methods). This allows for a detailed briefing of the whole team in charge at an early stage, and prompts pre-alert of the extracorporeal life support team and the catheterization lab according to local protocols if needed. This approach to history taking regularly leads to a situation in which the hospital team on the ward is better informed of the resuscitation circumstances and medical history than the delivering preclinical emergency team. We assume that this large amount of information about the patient and their arrest may at least partially explain our data. In situations where the resuscitation circumstances are less clear or history taking with witnesses is not possible, metabolic parameters may play a bigger role in early outcome estimation. Moreover, we acknowledge that small departments in particular may not be able to gather the same amount of information due to limited staff resources during the pre-alert phase.

### Outcome Models and External Validation

Various study groups developed and confirmed scores to predict outcome at an early stage. Balan and Seewald et al. built scores based on peri-arrest factors to predict mortality and good neurological outcome at hospital admission after OHCA (38, 39). Their validation cohorts reached areas under the receiver operating characteristics curves of 0.71 and 0.88. Our peri-arrest factors model (AUC 0.82)—although not created as a prediction score but as a reflection of clinical experience—lies within the range of these scores. The CAHP, OHCA, TTM, and C-GRApH models use both peri-arrest factors and metabolic parameters for the neurological outcome prediction after OHCA with areas under the curve of 0.91/0.85, 0.88, 0.82, and 0.81, respectively (13, 16, 17, 40). The recently published MIRACLE-2 score (consisting of peri-arrest factors plus blood pH levels) performed even better than the CAHP and OHCA scores, but showed similar performance in comparison with the TTM model (18).

In the estimation of neurological outcome after IHCA, the GO-FAR score was designed and evaluated with an AUC of 0.78 (41). Anderson et al. found that the addition of lactate levels to the intensive care score SAPS 3 improved the prognostic value in patients after cardiac arrest to an AUC of 0.80 (42). These performances are comparable to our IHCA model 2 (peri-arrest factors and metabolic parameters, AUC 0.779).

Although the most scores augmented their predictive value through the addition of metabolic parameters, no additive value for them was found in our data. We hypothesise that this is due to (i) higher accuracy in the raised peri-arrest factors in our cohort, (ii) different in- and exclusion criteria between the studies, and (iii) lack of external validation. Due to our standardised approach regarding the acquisition of peri-arrest factors (see “Setting”), we can assure the highest possible quality of peri-arrest factors. To our knowledge, this strategy is not widely established in other hospitals. According to that, the higher accuracy of peri-arrest factors in our data set may explain the lack of value in the addition of metabolic parameters. Second, the prediction scores mentioned above are not universally applicable due to the exclusion of several patient groups. As example, the MIRACLE-2 score only included patients with presumed cardiac origin of arrest; the CAHP score excluded all patients with drowning, intoxication or asphyxia. In our cohort, cardiac arrest patients irregarding of the presumed cause (with exception of trauma) are included. Third, most prediction scores are never validated externally (43). Thus, their predictive power is limited.

Although scores may help to identify patients with the most unfavourable outcome early, there is also a risk that patients with poor results may be ineffectively treated or denied further therapies. The same risk is inherent in the overestimation of supposedly poor metabolic parameters after admission.

Our prediction model is not designed to be used as an outcome score and was built for comparison purposes only. However, there are several possible implications for clinicians: First, peri-arrest factors can be collected when the patient is at the scene or during transportation to hospital, this allows for early resource allocation. Second, the peri-arrest factors can form a basis for answering prognosis based questions during the first conversation with relatives. In our opinion, relatives find it much easier to understand that, for example, unwitnessed arrests without basic life support measures are associated with bad outcome than they would understand numbers of metabolic parameters. With our model, we can give clinicians the confidence that peri-arrest factors provide sufficient information in this situation. Naturally blood gas analyses form a substantial part of post-resuscitation care and will play their role also in the future. However, our data show that peri-arrest factors suffice for early decision making and for first conversations about the presumed outcome.

### Strengths and Limitations

To reach a higher validity of the analysed data, we included both IHCA and OHCA to our calculations. This is in contrast to most other scores for outcome prediction, which were primarily designed for OHCA (17, 18, 38–40, 44). As only minor details were changed in the advanced life support guidelines over the last 15 years, we decided to include all patients from this long period in the analysis. This allowed us to study a high number of patients. The prospective inclusion and additional control of data quality in our resuscitation database ensures a high data quality within our sample.

Beside these strengths, we also faced some limitations. Due to the retrospective design of this study, we cannot exclude that both metabolic parameters and peri-arrest factors led to a self-fulfilling prophecy in outcome. It is known that patients with worse peri-arrest factors are treated less aggressively (45).

As we only included patients who already reached ROSC, our results may be not applicable to patients with ongoing CPR. In this particular group, metabolic parameters may play a more important role as they may act as surrogates for tissue perfusion and “resuscibility” of a patient. Porto and Jung showed that lactate levels prior to extracorporeal life support were associated with outcome (46, 47).

While outcome worsens with a longer duration of no flow and low flow time, we could not include the no flow interval in the peri-arrest factors prediction model without excluding non-witnessed patients in whom “no flow” times are not available.

We chose a more conservative significance level than in former papers because of the large sample size. Our conclusions were drawn based on this level and may differ from an approach using the regular alpha level of 0.05. For instance, the comparison of the AUC of model 1 and 2 in OHCA patients (*p* = 0.013) was considered not statistically significant. On the more conventional, but arbitrary level of 0.05, this *p*-value would fall below the treshhold. We believe that especially for hypothesis-generating, retrospective studies more conservative statistical approaches should be applied.

## Conclusion

Although the measurement of metabolic parameters in addition to peri-arrest factors may play a role in IHCA, no additional value in the prediction of good neurological outcome could be found in patients with OHCA. This highlights the importance of accurate anamnesis especially in patients with OHCA. Conversely, metabolic parameters may help to predict outcome in IHCA patients especially if peri-arrest factors are missing.

## Data Availability Statement

Derived data supporting the findings of this study are available from the corresponding author on reasonable request.

## Ethics Statement

The studies involving human participants were reviewed and approved by Ethics Committee of the Medical University of Vienna. Written informed consent for participation was not required for this study in accordance with the national legislation and the institutional requirements.

## Author Contributions

MM and JG: conceptualisation. MM, CC, CSchr, MP, MH, FS and FE: data curation. JG, CScho, and MM: methodology. FE and MH: supervision. MM: writing–original draft. JG, CScho, MS, CSchr, MP, CC, MK, FS, MH and FE: writing–review and editing. All authors contributed to the article and approved the submitted version.

## Conflict of Interest

The authors declare that the research was conducted in the absence of any commercial or financial relationships that could be construed as a potential conflict of interest.

## Publisher's Note

All claims expressed in this article are solely those of the authors and do not necessarily represent those of their affiliated organizations, or those of the publisher, the editors and the reviewers. Any product that may be evaluated in this article, or claim that may be made by its manufacturer, is not guaranteed or endorsed by the publisher.
