# Supplementary material for: Initial Blood pH, Lactate and Base Deficit Add No Value to Peri-Arrest Factors in Prognostication of Neurological Outcome After Out-of-Hospital Cardiac Arrest
Source: Front Med (Lausanne). 2021 Sep 16;8:697906. doi: 10.3389/fmed.2021.697906 (PMC8483260; doi:10.3389/fmed.2021.697906)
Supplement: Supplementary file 1 [file Data_Sheet_1.pdf]

Missing pattern figure, missing values map:

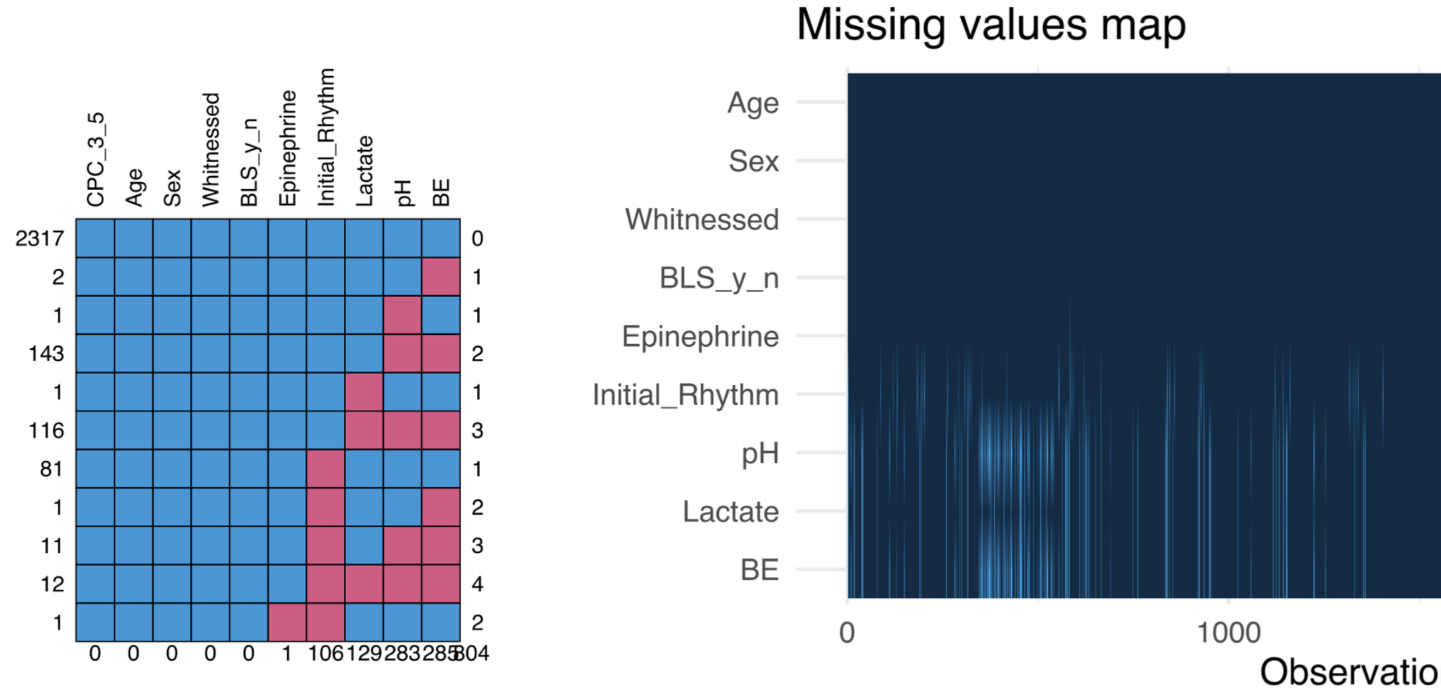

Models:

Model 1

overall (n=2317)

	Sig.	Exp(B)	99% C.I.for EXP(B)	
Age	.000	1.028	1.021	1.034
Sex	.131	1.182	.952	1.468
Witness status	.000	1.825	1.341	2.483
BLS	.000	2.189	1.705	2.809
Initial Rhythm	.000	3.466	2.846	4.220
Adrenaline	.000	1.464	1.391	1.540
Constant	.000			

OHCA

	Sig.	Exp(B)	99% C.I.for EXP(B)	
Age	.000	1.031	1.021	1.041
Sex	.080	1.258	.898	1.762
Witness status	.000	1.839	1.191	2.839
BLS	.000	2.042	1.447	2.881
Initial Rhythm	.000	4.007	2.935	5.471
Adrenaline	.000	1.492	1.384	1.609
Constant	.000			

IHCA

	Sig.	Exp(B)	99% C.I.for EXP(B)	
Age	.001	1.022	1.005	1.041
Sex	.972	.993	.573	1.720
Witness status	.902	1.066	.277	4.108
BLS	.146	3.536	.378	33.093
Initial Rhythm	.000	2.953	1.684	5.179
Adrenaline	.000	1.315	1.130	1.530
Constant	.000			

Model 2

overall (n=2317)

	Sig.	Exp(B)	99% C.I.for EXP(B)	
Age	.000	1.033	1.024	1.042
Sex	.235	1.145	.853	1.538
Witnessed	.001	1.729	1.143	2.616
BLS	.000	2.127	1.522	2.972
Initial Rhythm	.000	2.804	2.132	3.688
Adrenaline	.000	1.307	1.218	1.402
pH	.264	.563	.150	2.115
Lactate	.002	1.064	1.010	1.120
BE	.018	.966	.930	1.003
Constant	.916			

OHCA

	Sig.	Exp(B)	99% C.I.for EXP(B)	
Age	.000	1.036	1.026	1.047
Sex	.101	1.248	.881	1.766
Witnessed	.001	1.782	1.145	2.775
BLS	.000	2.040	1.437	2.897
Initial Rhythm	.000	3.190	2.294	4.435
Adrenaline	.000	1.345	1.242	1.457
pH	.574	.702	.139	3.544
Lactate	.009	1.065	1.001	1.134
BE	.103	.970	.925	1.018
Constant	.761			

IHCA

	Sig.	Exp(B)	99% C.I.for EXP(B)	
Age	.001	1.026	1.007	1.046
Sex	.570	.880	.494	1.569
Witnessed	.961	.974	.241	3.939
BLS	.130	3.721	.397	34.860
Initial Rhythm	.001	2.163	1.176	3.977
Adrenaline	.017	1.160	.988	1.361
pH	.187	.293	.027	3.220
Lactate	.064	1.068	.974	1.171
BE	.097	.960	.902	1.023
Constant	.391			

Model 3

overall (n=2317)

	Sig.	Exp(B)	99% C.I.for EXP(B)	
pH	.001	.223	.090	.551
Lactate	.000	1.116	1.077	1.157
BE	.012	.967	.942	.993
Constant	.004			

OHCA

	Sig.	Exp(B)	99% C.I.for EXP(B)	
pH	.023	.284	.068	1188
Lactate	.000	1.133	1.070	1.199
BE	.065	.970	.930	1.012
Constant	.049			

IHCA

	Sig.	Exp(B)	99% C.I.for EXP(B)	
pH	.013	.125	.015	1068
Lactate	.019	1.082	.992	1.180
BE	.083	.961	.906	1.020
Constant	.020			

pH

Overall

	Sig.	Exp(B)	99% C.I.for EXP(B)	
pH	.000	.012	.006	.024
Constant	.000			

OHCA

	Sig.	Exp(B)	99% C.I.for EXP(B)	
pH	.000	.011	.005	.026
Constant	.000			

IHCA

	Sig.	Exp(B)	99% C.I.for EXP(B)	
pH	.000	.013	.003	.060
Constant	.000			

Lactate

	Sig.	Exp(B)	99% C.I.for EXP(B)	
Lactate	.000	1.213	1.176	1.250
Constant	.000			

	Sig.	Exp(B)	99% C.I.for EXP(B)	
Lactate	.000	1.218	1.177	1.262
Constant	.000			

	Sig.	Exp(B)	99% C.I.for EXP(B)	
Lactate	.000	1.194	1.119	1.275
Constant	.000			

BE

	Sig.	Exp(B)	99% C.I.for EXP(B)	
BE	.000	.884	.867	.902
Constant	.000			

	Sig.	Exp(B)	99% C.I.for EXP(B)	
BE	.000	.881	.861	.902
Constant	.000			

	Sig.	Exp(B)	99% C.I.for EXP(B)	
BE	.000	.894	.860	.930
Constant	.000			

Variance inflation factor (VIF) values:

Variance Inflation Factor (VIF)

	overall		OHCA		IHCA	
	**Model 1**	**Model 2**	**Model 1**	**Model 2**	**Model 1**	**Model 2**
Age	1,033275	1,088946	1,040486	1,091617	1,040486	1,091617
Sex	1,026764	1,037321	1,035962	1,053828	1,035962	1,053828
Whitnessed	1,055741	1,053022	1,052662	1,049571	1,052662	1,049571
BLS	1,04622	1,045688	1,032545	1,033724	1,032545	1,033724
Epinephrine	1,016245	1,202335	1,015359	1,211541	1,015359	1,211541
Initial Rhythm	1,032581	1,101646	1,062224	1,142134	1,062224	1,142134
Lactate		2,724514		3,080838		3,080838
pH		2,930246		3,233254		3,233254
BE		3,21605		3,716224		3,716224
Maximum	3,716224					

Multiple imputations:

Significant codes:

*** = 0.001

** = 0.01

* = 0.05

IHCA, Model 1

1

**Coefficients:**	**Estimate**	**Std,**	**Error**	**z**	**value**
(Intercept)	-3,338896	0,47676	-7,003	2,50E-12	***
Age	0,027011	0,006603	4,091	4,30E-05	***
Sex	0,083878	0,198541	0,422	0,673	
Witnessed	0,378068	0,468933	0,806	0,42	
BLS_y_n	0,909376	0,709122	1,282	0,2	
Epinephrine	0,340697	0,057453	5,93	3,03E-09	***
Initial_Rhythm	1,426981	0,209415	6,814	9,48E-12	***

2

**Coefficients:**	**Estimate**	**Std,**	**Error**	**z**	**value**
(Intercept)	-3,381631	0,479801	-7,048	1,82E-12	***
Age	0,027716	0,006629	4,181	2,90E-05	***
Sex	0,059395	0,198802	0,299	0,765	
Witnessed	0,303895	0,468232	0,649	0,516	
BLS_y_n	0,939914	0,707689	1,328	0,184	
Epinephrine	0,343689	0,057504	5,977	2,28E-09	***
Initial_Rhythm	1,432547	0,209855	6,826	8,71E-12	***

IHCA, Model 1, mean

**Coefficients:**	**Estimate**	**z**	**value**
(Intercept)	-2,80976383	1,75E-12	***
Age	0,02291117	2,91E-05	***
Sex	0,06979167	5,62E-01	
Witnessed	0,26337333	4,17E-01	
BLS_y_n	0,77942483	1,55E-01	
Epinephrine	0,28587217	2,13E-09	***
Initial_Rhythm	1,1449036	9,36E-12	***

3	Coefficients:	Estimate	Std,	Error	z	value
	(Intercept)	-3,416447	0,481929	-7,089	1,35E-12	***
	Age	0,028138	0,006636	4,24	2,23E-05	***
	Sex	0,082989	0,19873	0,418	0,676	
	Witnessed	0,304684	0,468144	0,651	0,515	
	BLS_y_n	0,942231	0,707559	1,332	0,183	
	Epinephrine	0,341299	0,057395	5,946	2,74E-09	***
	Initial_Rhythm	1,460875	0,209644	6,968	3,21E-12	***

4	Coefficients:	Estimate	Std,	Error	z	value
	(Intercept)	-3,373397	0,480429	-7,022	2,19E-12	***
	Age	0,027065	0,006654	4,067	4,76E-05	***
	Sex	0,101413	0,199711	0,508	0,612	
	Witnessed	0,290584	0,468755	0,62	0,535	
	BLS_y_n	0,962479	0,708625	1,358	0,174	
	Epinephrine	0,340268	0,05749	5,919	3,24E-09	***
	Initial_Rhythm	1,482003	0,209292	7,081	1,43E-12	***

5	Coefficients:	Estimate	Std,	Error	z	value
	(Intercept)	-3,348212	0,478585	-6,996	2,63E-12	***
	Age	0,027537	0,006626	4,156	3,24E-05	***
	Sex	0,091075	0,198343	0,459	0,646	
	Witnessed	0,303009	0,466357	0,65	0,516	
	BLS_y_n	0,922549	0,705658	1,307	0,191	
	Epinephrine	0,34928	0,057767	6,046	1,48E-09	***
	Initial_Rhythm	1,382987	0,207621	6,661	2,72E-11	***

IHCA, Model 2

	Coefficients:	Estimate	Std,	Error	z	value
	(Intercept)	1,431594	6,616048	0,216	0,82869	
	Age	0,032362	0,00714	4,533	5,83E-06	***
	Sex	-0,002191	0,20856	-0,011	0,991618	
	Witnessed	0,34661	0,478292	0,725	0,468646	
1	BLS_y_n	0,900405	0,690748	1,304	0,192397	
	Epinephrine	0,2089	0,06044	3,456	0,000548	***
	Initial_Rhythm	1,165994	0,221819	5,257	1,47E-07	***
	pH	-0,775855	0,902559	-0,86	0,39	
	Lactate	0,09405	0,034047	2,762	0,005738	**
	BE	-0,034941	0,023413	-1,492	0,135593	

	Coefficients:	Estimate	Std,	Error	z	value
	(Intercept)	-0,810904	6,435035	-0,126	0,899721	
	Age	0,030784	0,007151	4,305	1,67E-05	***
	Sex	0,004827	0,208756	0,023	0,981552	
	Witnessed	0,32304	0,481106	0,671	0,501932	
2	BLS_y_n	0,983466	0,702869	1,399	0,161748	
	Epinephrine	0,219781	0,060161	3,653	0,000259	***
	Initial_Rhythm	1,235538	0,222659	5,549	2,87E-08	***
	pH	-0,467226	0,877236	-0,533	0,594303	
	Lactate	0,085716	0,033621	2,549	0,010789	*
	BE	-0,046932	0,023074	-2,034	0,041952	*

IHCA, Model 2, mean

Coefficients:	Estimate	z	value
(Intercept)	0,30320367	8,49E-01	
Age	0,02640683	1,01E-05	***
Sex	0,0011885	9,63E-01	
Witnessed	0,298513	4,58E-01	
BLS_y_n	0,76194283	1,88E-01	
Epinephrine	0,179623	3,75E-04	***
Initial_Rhythm	0,98574433	2,33E-07	***
pH	-0,52529433	4,78E-01	
Lactate	0,07369883	9,36E-03	**
BE	-0,035786	7,16E-02	

3	Coefficients:	Estimate	Std,	Error	z	value
	(Intercept)	0,73017	6,399293	0,114	0,909157	
	Age	0,031024	0,007142	4,344	1,40E-05	***
	Sex	0,020087	0,208564	0,096	0,923275	
	Witnessed	0,455475	0,48797	0,933	0,350609	
	BLS_y_n	0,872074	0,695739	1,253	0,210042	
	Epinephrine	0,215633	0,059964	3,596	0,000323	***
	Initial_Rhythm	1,139979	0,22344	5,102	3,36E-07	***
	pH	-0,672499	0,872775	-0,771	0,440985	
	Lactate	0,088778	0,033996	2,611	0,009017	**
	BE	-0,043221	0,023284	-1,856	0,063419	

4	Coefficients:	Estimate	Std,	Error	z	value
	(Intercept)	1,80597	6,168646	0,293	0,769701	
	Age	0,031906	0,007139	4,469	7,85E-06	***
	Sex	0,002678	0,208176	0,013	0,989735	
	Witnessed	0,370808	0,476823	0,778	0,436767	
	BLS_y_n	0,880048	0,687043	1,281	0,200221	
	Epinephrine	0,218599	0,060474	3,615	0,000301	***
	Initial_Rhythm	1,119662	0,224714	4,983	6,27E-07	***
	pH	-0,827327	0,841853	-0,983	0,325733	
	Lactate	0,089114	0,033842	2,633	0,008457	**
	BE	-0,040322	0,023183	-1,739	0,081977	

	Coefficients:	Estimate	Std,	Error	z	value
	(Intercept)	-1,337608	6,442898	-0,208	0,835534	
	Age	0,032365	0,007162	4,519	6,22E-06	***
	Sex	-0,01827	0,209196	-0,087	0,930405	
	Witnessed	0,295145	0,474483	0,622	0,533919	
**5**	BLS_y_n	0,935664	0,688156	1,36	0,173934	
	Epinephrine	0,214825	0,061174	3,512	0,000445	***
	Initial_Rhythm	1,253293	0,225195	5,565	2,62E-08	***
	pH	-0,408859	0,87679	-0,466	0,640991	
	Lactate	0,084535	0,033957	2,489	0,012792	*
	BE	-0,0493	0,023394	-2,107	0,035081	*

OHCA, Model 1						
	**Coefficients:**	**Estimate**	**Std,**	**Error**	**z**	**value**
				-		
	(Intercept)	-3,25934	0,23553	13,838	2,00E-16	***
	Age	0,02852	0,00345	8,267	2,00E-16	***
**1**	Sex	0,17989	0,12216	1,473	0,140869	
	Witnessed	0,56208	0,15836	3,549	0,000386	***
	BLS_y_n	0,76678	0,12872	5,957	2,57E-09	***
	Epinephrine	0,43411	0,02797	15,518	2,00E-16	***
	Initial_Rhythm	1,46096	0,11286	12,945	2,00E-16	***

	**Coefficients:**	**Estimate**	**Std,**	**Error**	**z**	**value**
				-		
	(Intercept)	-3,262161	0,235399	13,858	2,00E-16	***
	Age	0,028844	0,003447	8,369	2,00E-16	***
**2**	Sex	0,182128	0,121774	1,496	0,134753	
	Witnessed	0,583575	0,157995	3,694	0,000221	***
	BLS_y_n	0,763614	0,128388	5,948	2,72E-09	***
	Epinephrine	0,433195	0,027887	15,534	2,00E-16	***
	Initial_Rhythm	1,420253	0,112594	12,614	2,00E-16	***

	**Coefficients:**	**Estimate**	**Std,**	**Error**	**z**	**value**
				-		
	(Intercept)	-3,250603	0,235193	13,821	2,00E-16	***
	Age	0,028389	0,003445	8,24	2,00E-16	***
**3**	Sex	0,172159	0,12192	1,412	0,157932	
	Witnessed	0,571058	0,158481	3,603	0,000314	***
	BLS_y_n	0,771998	0,128509	6,007	1,89E-09	***
	Epinephrine	0,436107	0,027977	15,588	2,00E-16	***
	Initial_Rhythm	1,439857	0,112654	12,781	2,00E-16	***

OHCA, Model 1, mean			
**Coefficient**	**Estimate**	**z**	**value**
(Intercept)	-3,248252	2,00E-16	***
Age	0,0284072	2,40E-16	***
Sex	0,1805986	1,39E-01	
Witnessed	0,5670806	3,45E-04	***
BLS_y_n	0,7701818	2,10E-09	***
Epinephrine	0,435103	2,00E-16	***
Initial_Rhythm	1,4336764	2,00E-16	***

	Coefficients:	Estimate	Std,	Error	z	value
				-		
	(Intercept)	-3,238782	0,234628	13,804	2,00E-16	***
	Age	0,028199	0,003436	8,208	2,25E-16	***
**4**	Sex	0,18798	0,121692	1,545	0,122414	
	Witnessed	0,559759	0,158009	3,543	0,000396	***
	BLS_y_n	0,778717	0,128277	6,071	1,27E-09	***
	Epinephrine	0,437088	0,027962	15,631	2,00E-16	***
	Initial_Rhythm	1,413385	0,112184	12,599	2,00E-16	***

	Coefficients:	Estimate	Std,	Error	z	value
				-		
	(Intercept)	-3,230374	0,23506	13,743	2,00E-16	***
	Age	0,028084	0,003448	8,146	3,77E-16	***
**5**	Sex	0,180836	0,121937	1,483	0,138067	
	Witnessed	0,558931	0,158106	3,535	0,000408	***
	BLS_y_n	0,7698	0,12845	5,993	2,06E-09	***
	Epinephrine	0,435015	0,027972	15,552	2,00E-16	***
	Initial_Rhythm	1,433927	0,112409	12,756	2,00E-16	***

OHCA, Model 2

1

Coefficients:	Estimate	Std,	Error	z	value
(Intercept)	-5,503962	4,315838	-1,275	0,202205	
Age	0,03366	0,003653	9,215	2,00E-16	***
Sex	0,191594	0,125698	1,524	0,127446	
Witnessed	0,521766	0,161691	3,227	0,001251	**
BLS_y_n	0,761317	0,131182	5,804	6,49E-09	***
Epinephrine	0,322622	0,029478	10,945	2,00E-16	***
Initial_Rhythm	1,181908	0,118701	9,957	2,00E-16	***
pH	0,175882	0,585546	0,3	0,763892	
Lactate	0,084105	0,022258	3,779	0,000158	***
BE	-0,03794	0,01721	-2,205	0,027488	*

2

Coefficients:	Estimate	Std,	Error	z	value
(Intercept)	-5,434451	4,359792	-1,246	0,212583	
Age	0,032856	0,003632	9,047	2,00E-16	***
Sex	0,171733	0,126074	1,362	0,173149	
Witnessed	0,503512	0,161676	3,114	0,001844	**
BLS_y_n	0,769428	0,131074	5,87	4,35E-09	***
Epinephrine	0,328206	0,029569	11,1	2,00E-16	***
Initial_Rhythm	1,207661	0,118804	10,165	2,00E-16	***
pH	0,175306	0,590868	0,297	0,766701	
Lactate	0,083468	0,022023	3,79	0,000151	***
BE	-0,034355	0,017368	-1,978	0,047919	*

OHCA, Model 2 mean

Coefficients:	Estimate	z	value
(Intercept)	-5,275805	2,22E-01	
Age	0,0335686	2,00E-16	***
Sex	0,1798832	1,54E-01	
Witnessed	0,5161356	1,45E-03	**
BLS_y_n	0,7634572	6,19E-09	***
Epinephrine	0,3264038	2,00E-16	***
Initial_Rhythm	1,2175584	2,00E-16	***
pH	0,146535	8,03E-01	
Lactate	0,0849706	1,45E-04	***
BE	-0,0338138	5,43E-02	

	Coefficients:	Estimate	Std,	Error	z	value
3	(Intercept)	-4,779305	4,335211	-1,102	0,27027	
	Age	0,034147	0,003652	9,349	2,00E-16	***
	Sex	0,180091	0,126319	1,426	0,15396	
	Witnessed	0,501477	0,161747	3,1	0,00193	**
	BLS_y_n	0,760387	0,131635	5,776	7,63E-09	***
	Epinephrine	0,328062	0,029364	11,172	2,00E-16	***
	Initial_Rhythm	1,251452	0,119545	10,468	2,00E-16	***
	pH	0,073013	0,588022	0,124	0,90118	
	Lactate	0,091123	0,02241	4,066	4,78E-05	***
	BE	-0,027228	0,017202	-1,583	0,11345	

	Coefficients:	Estimate	Std,	Error	z	value
4	(Intercept)	-5,39849	4,312869	-1,252	0,210673	
	Age	0,033938	0,003655	9,285	2,00E-16	***
	Sex	0,183731	0,125867	1,46	0,144367	
	Witnessed	0,524753	0,161866	3,242	0,001187	**
	BLS_y_n	0,759733	0,131461	5,779	7,51E-09	***
	Epinephrine	0,323343	0,029485	10,966	2,00E-16	***
	Initial_Rhythm	1,237249	0,119067	10,391	2,00E-16	***
	pH	0,159377	0,584947	0,272	0,785266	
	Lactate	0,084215	0,022275	3,781	0,000156	***
	BE	-0,035512	0,017309	-2,052	0,040198	*

	Coefficients:	Estimate	Std,	Error	z	value
	(Intercept)	-5,262817	4,238983	-1,242	0,21441	

**5**	Age	0,033242	0,003641	9,129	2,00E-16	***
	Sex	0,172267	0,125781	1,37	0,17082	
	Witnessed	0,52917	0,161441	3,278	0,00105	**
	BLS_y_n	0,766421	0,131053	5,848	4,97E-09	***
	Epinephrine	0,329786	0,029612	11,137	2,00E-16	***
	Initial_Rhythm	1,209522	0,118986	10,165	2,00E-16	***
	pH	0,149097	0,575262	0,259	0,7955	
	Lactate	0,081942	0,022104	3,707	0,00021	***
	BE	-0,034034	0,016784	-2,028	0,04258	*

[redacted]

Multiple Imputations, AUC

	IHCA, Model 1		IHCA, Model 2				
Imputation	AUC	CI 99%	AUC	CI 99%	**Diff AUC**	**p-value**	
LogReg 1	0,778	0,7293-0,8266	0,815	0,7696-0,8595	0,037	< 0.001	*
LogReg 2	0,78	0,7316-0,8286	0,816	0,7717-0,8613	0,036	< 0.001	*
LogReg 3	0,779	0,7307-0,8279	0,815	0,7704-0,8602	0,036	< 0.001	*
LogReg 4	0,782	0,7338-0,8304	0,815	0,7694-0,8596	0,033	< 0.001	*
LogReg 5	0,778	0,7289-0,8264	0,817	0,7724-0,862	0,039	< 0.001	*
**Average**	0,7794		0,8156		0,0362	< 0.001	*

	OHCA, Model 1		OHCA, Model 2				
Imputation	AUC	CI 99%	AUC	CI 99%	**Diff AUC**	**p-value**	
LogReg 1	0,836	0,8136-0,8586	0,845	0,8228-0,8663	0,009	0,003	
LogReg 2	0,834	0,8117-0,857	0,844	0,8223-0,8659	0,01	0,002	*
LogReg 3	0,835	0,8126-0,8577	0,846	0,8247-0,868	0,011	0,001	*
LogReg 4	0,834	0,8116-0,8569	0,846	0,8242-0,8675	0,012	0,0025	
LogReg 5	0,835	0,8122-0,8575	0,844	0,8219-0,8655	0,009	0,007	
**Average**	0,835		0,845		0,010	0,003	

Bonferroni correction:

p = 0.01 -> p_corr = 0.002

* significant after Bonferroni correction

IHCA LogReg 1	
	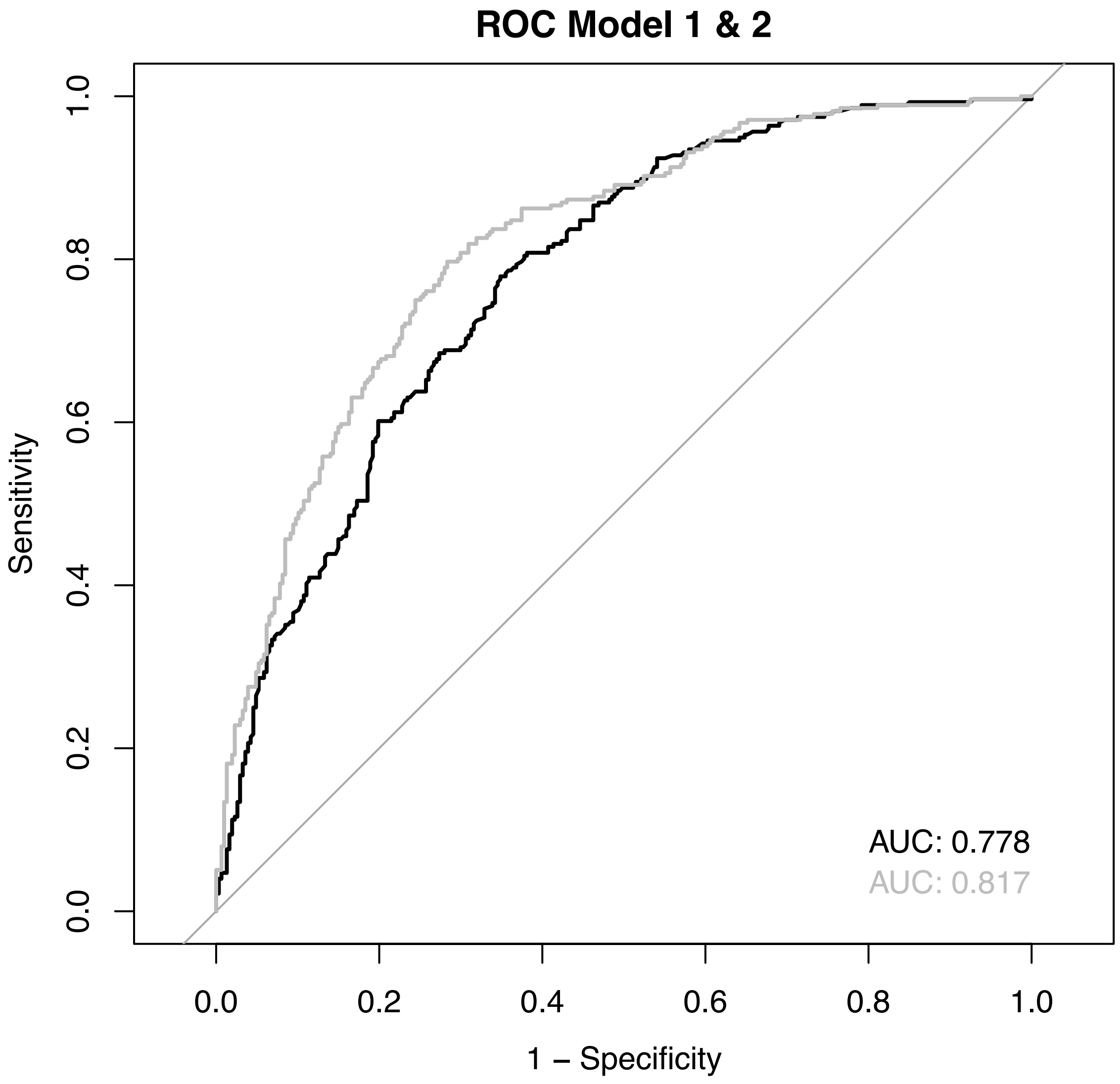

**IHCA LogReg 2**	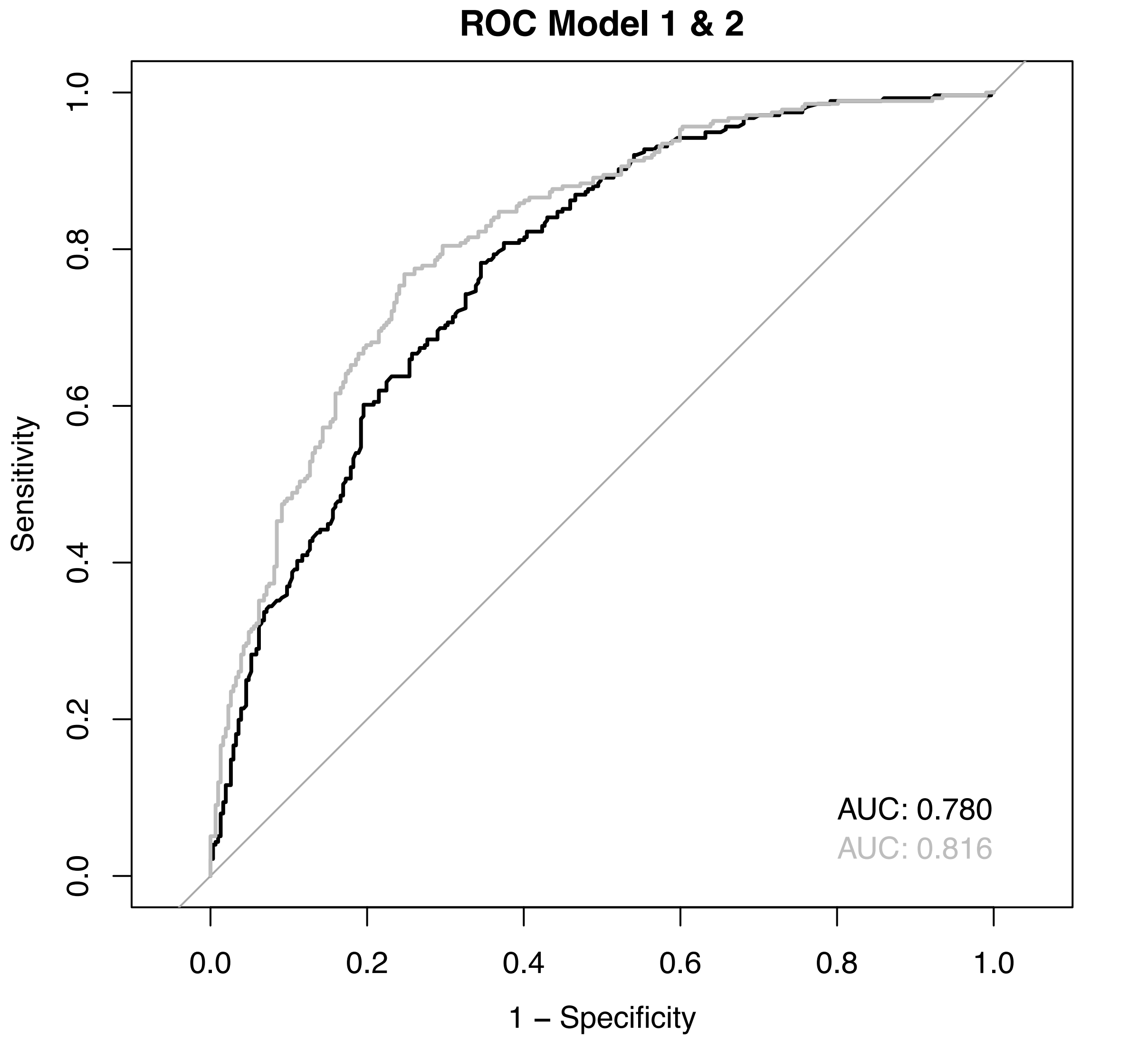

IHCA LogReg 3	
**IHCA LogReg 3**	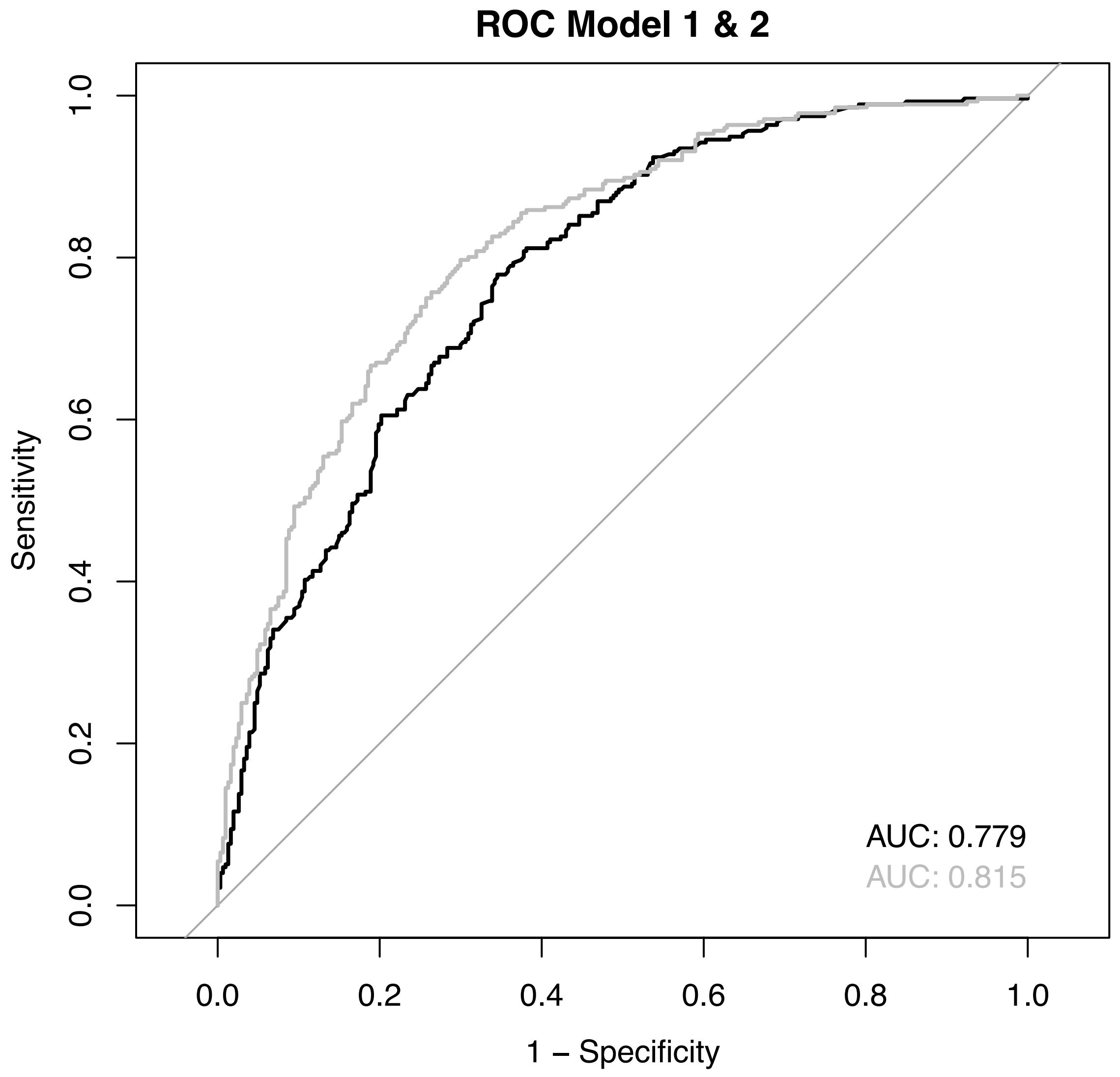

**IHCA LogReg 4**	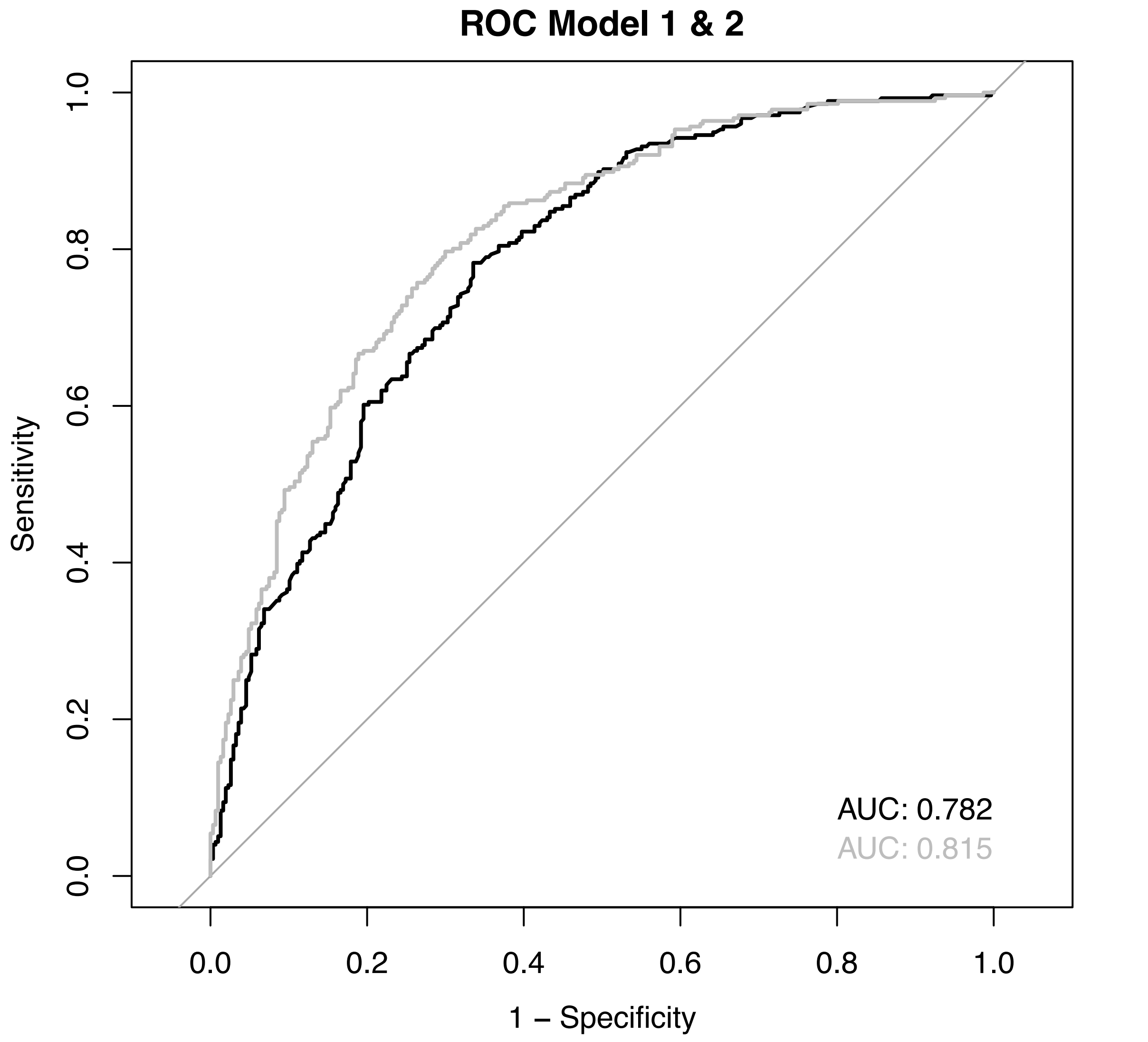

IHCA LogReg 5	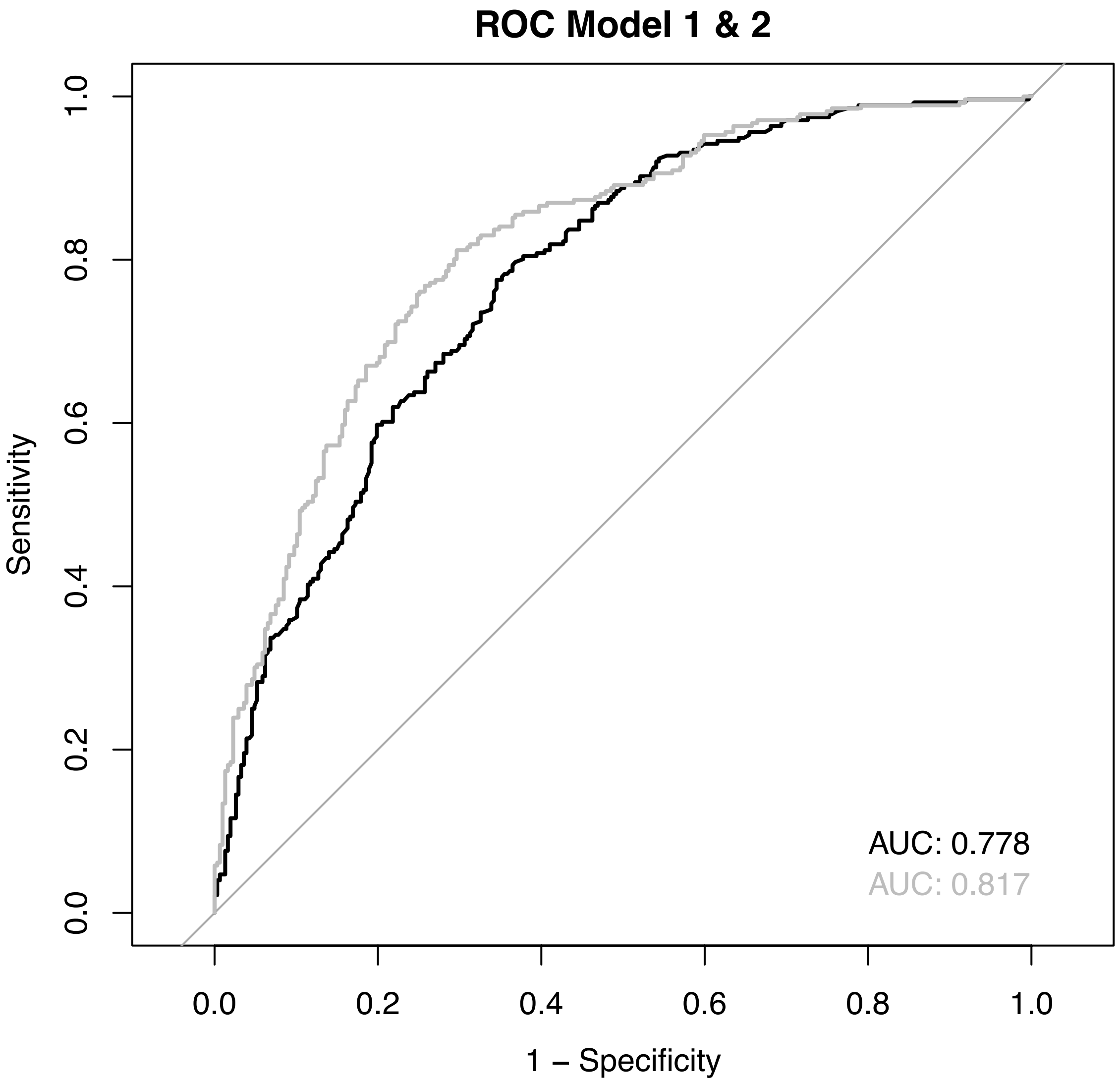

OHCA LogReg 1	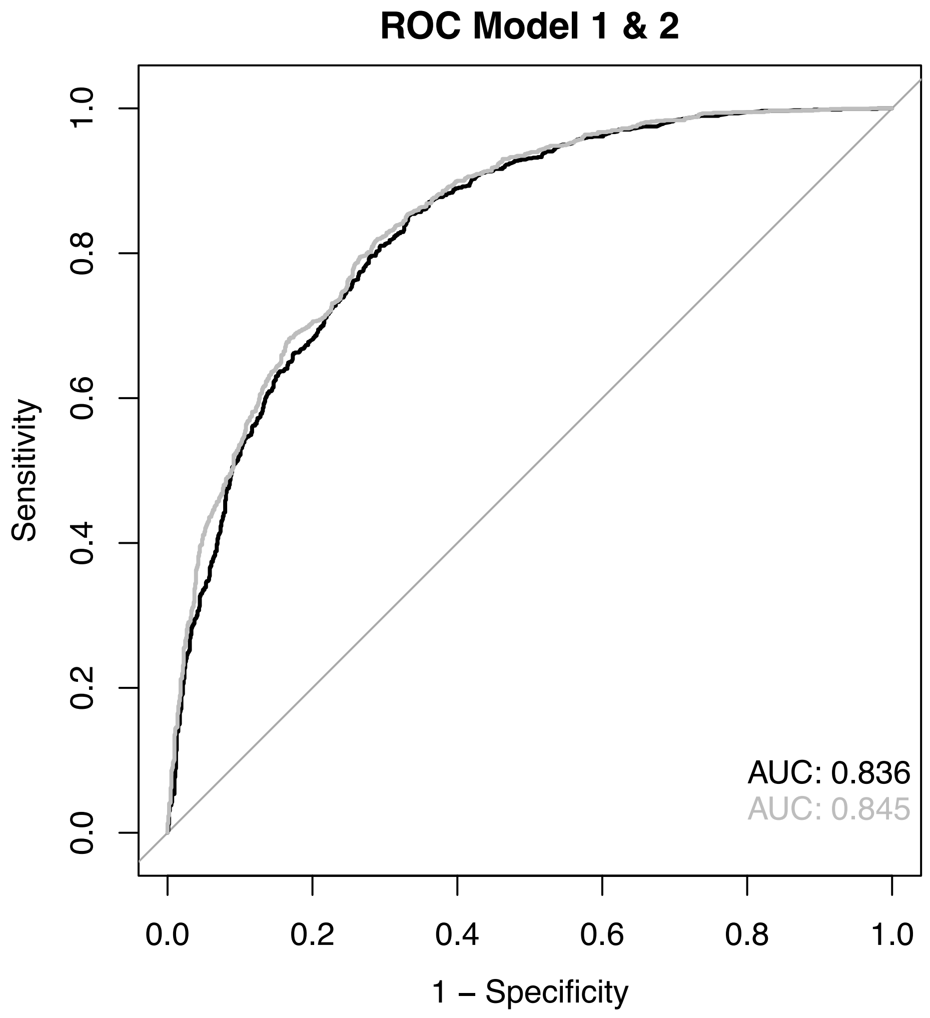

**OHCA LogReg 2**	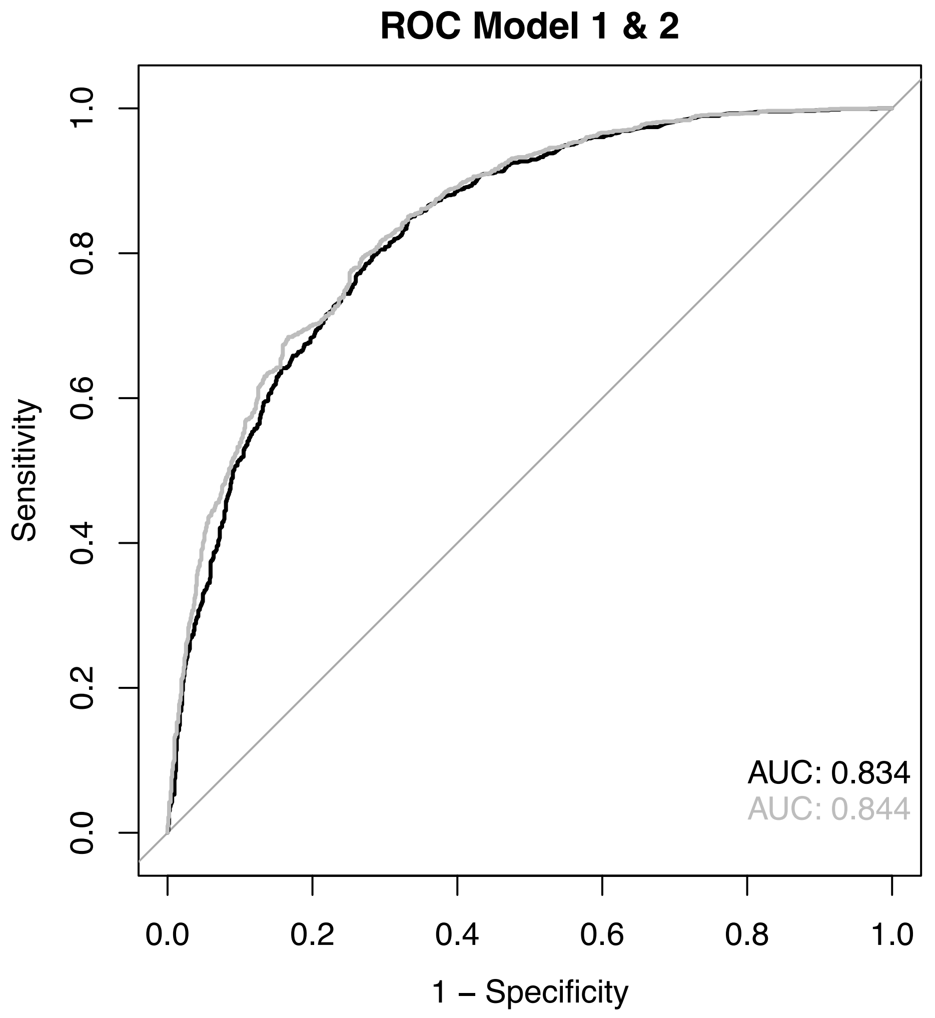

**OHCA LogReg 3**	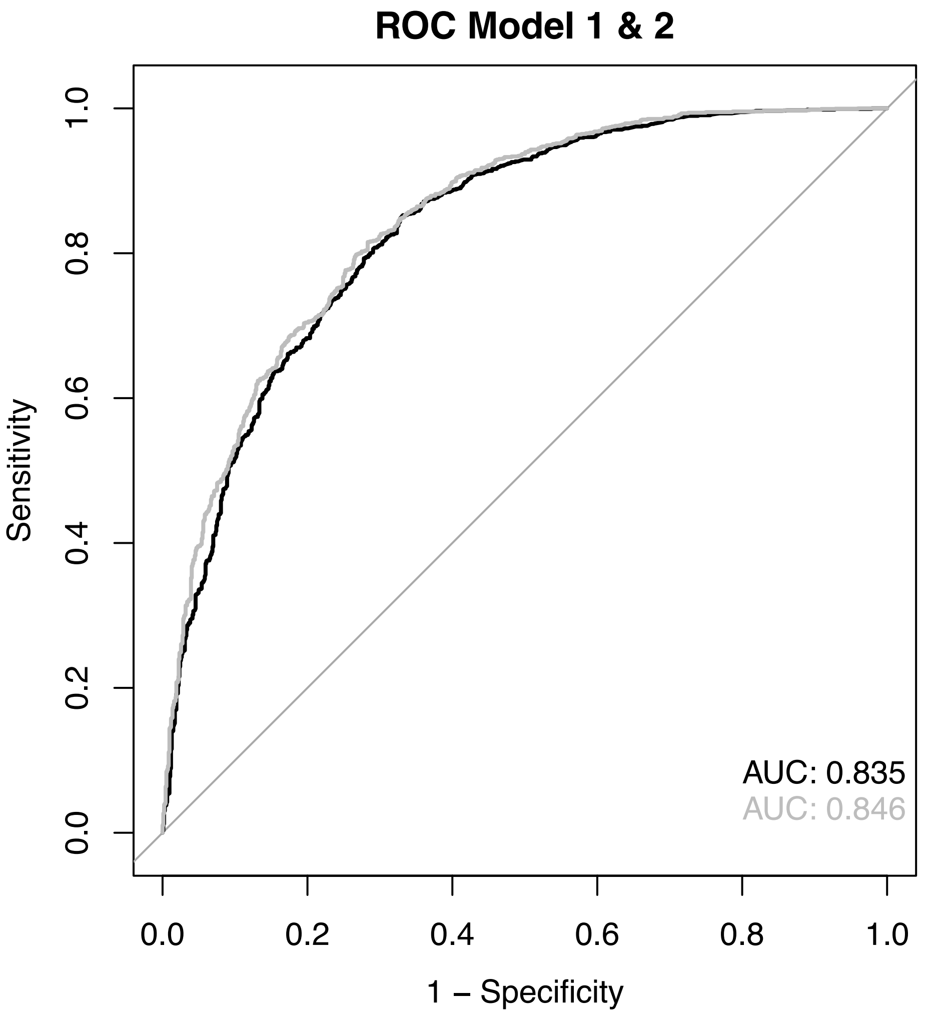

OHCA LogReg 4	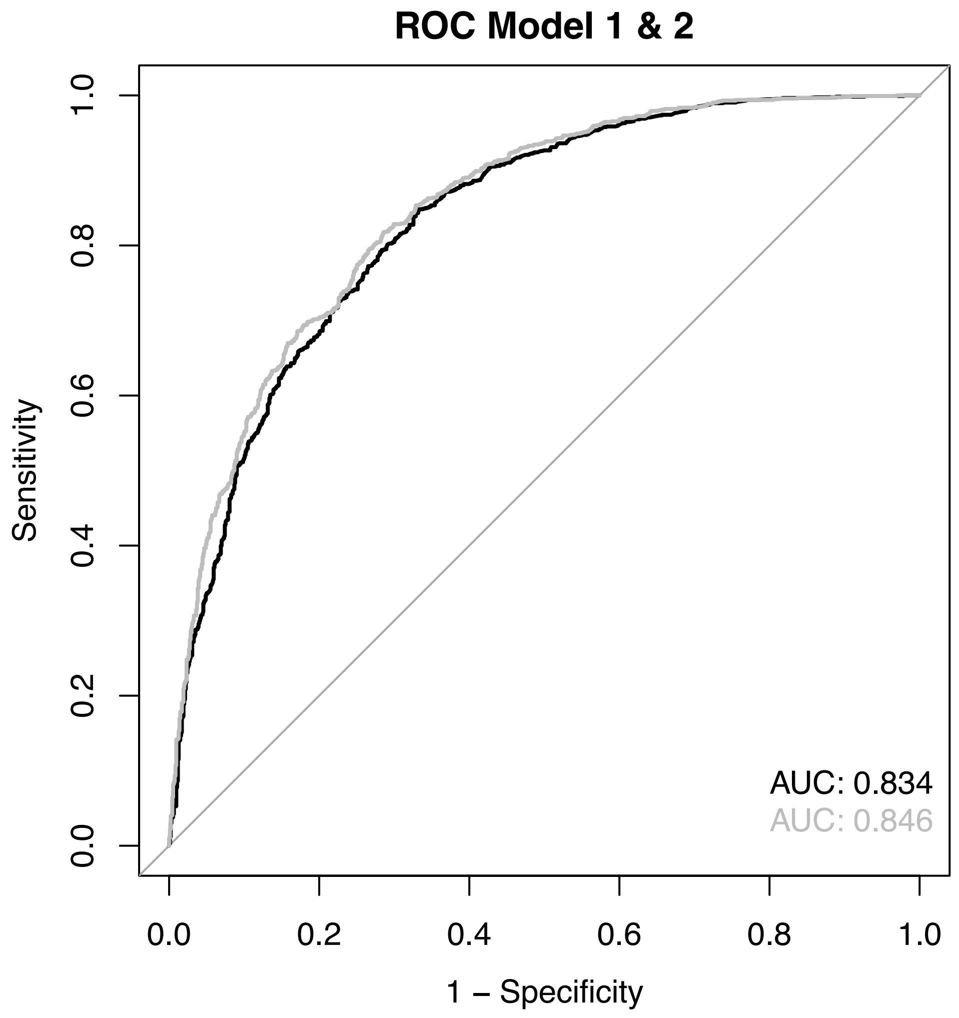

**OHCA LogReg 5**	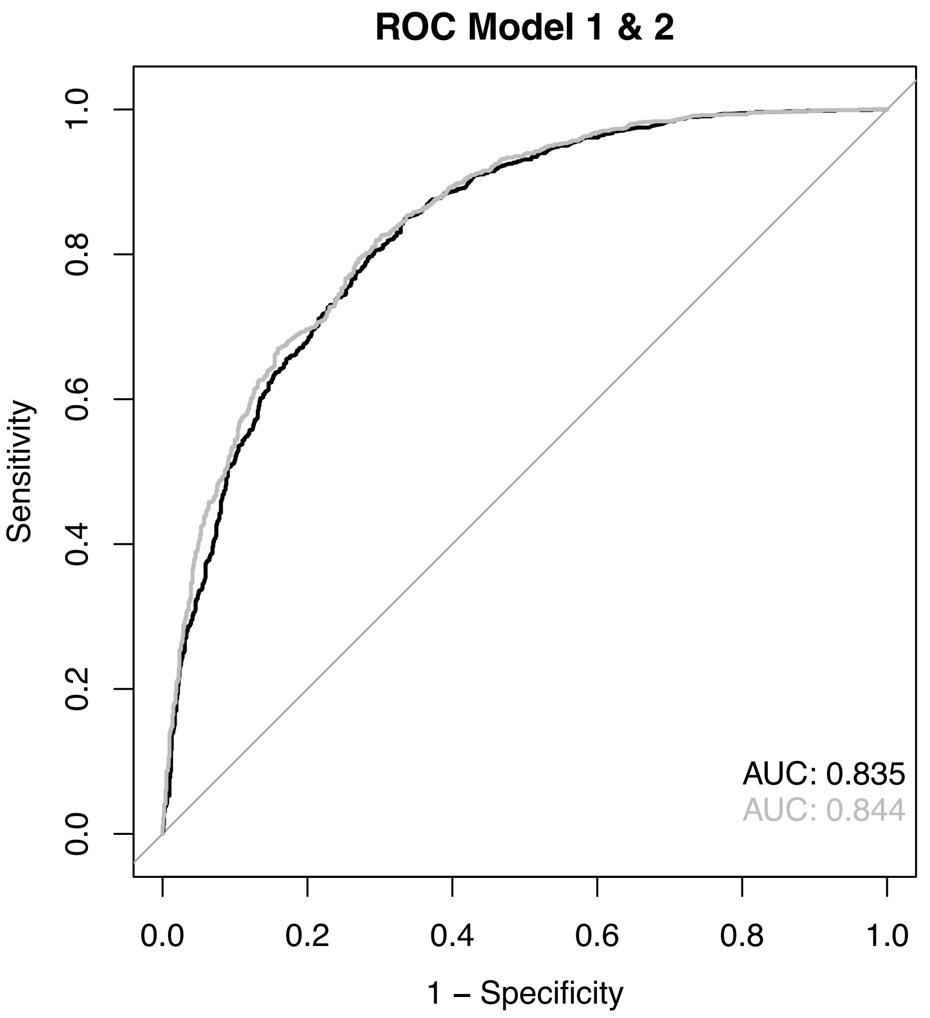
